# Effects of cognitive behavioral therapy on cognition, neuropsychiatric symptoms, and quality of life in Alzheimer’s disease: a meta-analysis

**DOI:** 10.3389/fpsyt.2025.1648225

**Published:** 2025-09-29

**Authors:** Yuxin Gao, Jiajun Yin, Yuqin Sun, Ting Li, Yue Wu

**Affiliations:** ^1^ Department of Psychiatry, The Affiliated Mental Health Center of Jiangnan University, Wuxi, China; ^2^ Department of Psychiatry, Wuxi School of Medicine, Jiangnan University, Wuxi, China; ^3^ Brain Science Basic Laboratory, The Affiliated Mental Health Center of Jiangnan University, Wuxi, China

**Keywords:** Alzheimer’s disease, cognitive behavioral therapy, cognition function, neuropsychiatric symptoms, systematic review, meta-analysis, quality of life

## Abstract

**Background:**

Alzheimer’s disease (AD) is a progressive neurodegenerative disorder, often accompanied by cognitive decline and neuropsychiatric symptoms, which substantially impair patients’ quality of life. Cognitive behavioral therapy (CBT) has shown effects in improving mood and quality of life in depression and anxiety, but systematic evidence of its application in AD is still limited.

**Objective:**

This study aims to systematically assess the efficacy of CBT interventions on global cognition, neuropsychiatric symptoms (including depression severity) and Quality of Life (QoL) in AD.

**Method:**

PubMed, Embase, Cochrane, and Web of Science databases were researched from the inception of each database to March 2025. The search strategy included MESH terms “Alzheimer Disease” and “Cognitive Behavioral Therapy”, combined with the Boolean operator “AND”. Randomized controlled trials involving CBT-adapted protocols were included. Data were pooled using random-effects models (Hedges’ g with 95%CIs), with subgroup analyses by intervention duration. Meta-analyses were performed using Stata MP 18.0, with bias risk assessed by the Cochrane Risk of Bias tool RevMan 5.4.

**Result:**

A total of 15 randomized controlled trials (n=2,135 participants) were included. The meta-analysis showed that CBT significantly improved global cognition (MMSE: SMD = 0.67, 95%CI=0.31 to 1.02, p<0.001), though heterogeneity was high (I²=86.9%). No significant effects were observed for neuropsychiatric symptoms, or QoL. Subgroup analyses revealed medium-term interventions (8–16 weeks) reduced depressive symptoms and improved QoL, while long-term CBT (>16 weeks) enhanced cognition.

**Conclusions:**

CBT demonstrates significant benefits for global cognition in AD, with medium-term interventions effective for mood and QoL outcomes. These findings support CBT intervention as viable non-pharmacological strategies for AD management, though methodological limitations and considerable heterogeneity warrant cautious interpretation.

## 1 Introduction

Alzheimer’s disease (AD) is a major type of dementia and poses a growing global health burden (1). Population aging and growth are projected to drive further rises, with global dementia cases expected to grow from 57 million in 2019 to 152 million by 2050 (2). As the predominant subtype of dementia (accounting for 60–80% of cases) and the most common neurodegenerative disorder, AD ranks among this century’s most lethal, costly, and debilitating diseases (3). Its core features include progressive cognitive decline (involving memory, language, and other domains), which severely impairs daily functioning, along with a spectrum of neuropsychiatric symptoms such as depression, agitation, apathy, anxiety, and psychotic features (4, 5). These multidimensional impairments not only accelerate disease progression and increase caregiver burden but also highlight the urgent need for effective therapeutic strategies.

Recent years, disease-modifying treatments such as donanemab and lecanemab for AD have been approved by the Food and Drug Administration (6). However, the effects of these treatments remain limited by multiple factors, including the stage of the disease, potential side effects, cost implications, and individual variations (7). Therefore, non-pharmacological strategies that can delay the progressive functional deterioration in AD may be a reasonable alternative, encompassing approaches such as music therapy, exercise interventions, repetitive transcranial magnetic stimulation (rTMS) and cognitive interventions (8). As a specific form of cognitive intervention, Cognitive Behavioral Therapy (CBT) focuses on dynamic interaction between cognitive processes, emotional responses, and behavioral patterns (9). CBT was initially mainly applied in the treatment of mood disorders such as depression and anxiety (10). Studies demonstrated that individuals suffering from dementia can learn and develop skills (11). This suggests that CBT, as an effective psychological intervention method, can be introduced into the treatment and care of AD (12, 13). In the context of the ongoing progress in interdisciplinary research integrating geriatrics, neurology, and psychology, the application of CBT in AD gradually entered a more clinically refined stage (14). In non-pharmacological treatment strategies for AD, CBT is often integrated with other interventions such as cognitive training, mindfulness and home-based occupational therapy, forming comprehensive and multifaceted intervention programs (15–17).

However, the empirical evidence supporting CBT’s efficacy in AD has been mixed. Systematic reviews have yielded inconclusive findings. For instance, a 2022 review pooling people living with dementia (PLWD) across etiologies found psychological therapies (often including CBT components) reduce depression and anxiety, but effects on global cognition, activities of daily living, and quality of life remain inconsistent (18). Earlier reviews of “cognitive therapy” in dementia (spanning multiple modalities beyond CBT) also judged the evidence inconclusive due to variability in interventions and outcomes (19). Therefore, the present meta-analysis aims to systematically evaluate the effects of CBT on cognition, neuropsychiatric symptoms, and quality of life in patients with clinically diagnosed Alzheimer’s disease, using data from randomized controlled trials (RCTs).

## 2 Materials and methods

### 2.1 Literature search

This meta-analysis review adheres to the guidelines of PRISMA 2020 (53) and has been registered in PROSPERO database (Registration ID: CRD420251056987).

Following the PICOS framework (20), we systematically searched PubMed, Embase, Cochrane library, and Web of Science databases from inception to March 2025. In PubMed, the search strategy combined Medical Subject Headings (MeSH) and free-text terms for both Alzheimer’s disease and cognitive behavioral therapy, which were linked using the Boolean operator AND. Similar combinations of subject words and free words were applied to other databases to ensure comprehensive retrieval. The complete search strategy is provided in **Supplementary Method S1**.

### 2.2 Inclusion and exclusion criteria

Included studies were required to meet the following criteria:

- Patients: Older individuals diagnosed with Alzheimer’s disease (AD). There were no restrictions regarding age, sex, or race.
- Different types of Randomized Controlled Trial included cognitive interventions that conform to the core mechanism of CBT (21) vs. placebo, usual standard clinical care, Baseline treatments.
- Studies were required to report at least one target outcome. The primary outcomes were defined as post-intervention scores in: (a) global cognitive function assessed by the Mini-Mental State Examination (MMSE) (22); (b) depression severity measured using the Cornell Scale for Depression in Dementia (CSDD) (23)or Geriatric Depression Scale (GDS) (24); (c) behavioral and psychological symptoms evaluated with the Neuropsychiatric Inventory (NPI) (25); and(d) subjective well-being quantified through quality of life (QoL) instrument (26).
- Published in English or Spanish up to March 27, 2025.

Excluded studies had to meet the following criteria:

- Other types of dementia.
- No available data.
- Case Report or Conference Summary.
- Literature that has been repeatedly published by the same author or contains duplicate data.

The inclusion and exclusion criteria were organized into a PICOS table (**Table 1**) for ease of understanding.

**Table 1:** PICOS criteria for study selection.

Element	Description
Population	Patients diagnosed with Alzheimer’s disease according to the NINCDS-ADRDA criteria, DSM-IV or ICD-10 diagnostic criteria were included. Individuals with other types of dementia or mild cognitive impairment (MCI) were excluded.
Intervention	The included intervention was required to align with core CBT principles by incorporating at least one of the following components: cognitive restructuring, behavioral activation, problem-solving training, psychoeducation, reception-based cognitive intervention, stress management through relaxation or mindfulness.
Comparator	Control groups consisted of placebo, usual standard clinical care, or baseline treatments.
Outcomes	The primary outcomes were defined as post-intervention scores in global cognitive function assessed by MMSE; depression severity measured using CSDD or GDS; behavioral and psychological symptoms evaluated with NPI; and subjective well-being quantified QoL. Study was required to report at least one target outcome and were excluded if means and standard deviations could not be extracted.
Study design	The study type was RCT.

Studies for this systematic review and meta-analysis were screened according to the criteria listed in the table. MMSE, Mini-Mental State Examination; CSDD, Cornell Scale for Depression in Dementia; GDS, Geriatric Depression Scale; NPI, Neuropsychiatric Inventory; QoL, quality of life; RCT, Randomized Controlled Trial.

### 2.3 Data extraction

**Figure 1:**
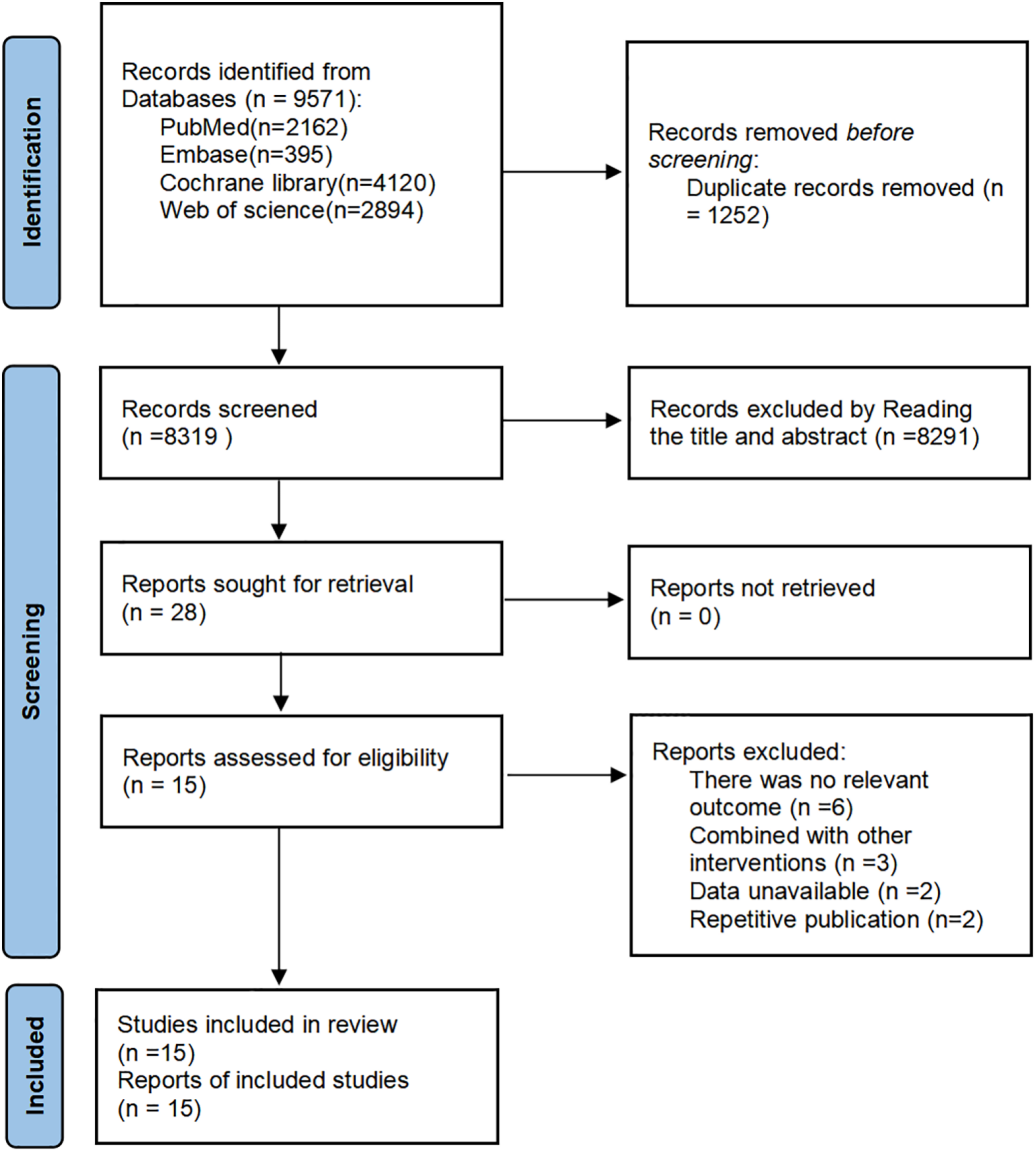
PRISMA (2020) flow chart of the study selection process (53).

The selection of studies was independently selected by two reviewers (YG and YS). The screening was conducted based on title and abstract, and those that meet the established criteria are included. Then the data of studies including Author, year of publication, country, characteristics of the participants (sample size, male/female, mean age), intervention, duration and outcomes were extracted by two reviewers (YG and YS), followed by the extracted data cross-checked. If there were any discrepancies, a third reviewer (TL) would be appointed to make the final decision. Finally, the unambiguous data was incorporated into the dataset. The study selection process is illustrated in the PRISMA 2020 flow diagram (**Figure 1**).

### 2.4 Subgroup classification and justification

In this meta-analysis, CBT duration was categorized into short-term (1–8 weeks), medium-term (8–16 weeks), and long-term phases (beyond 16 weeks). Based on existing literature, CBT interventions typically range from 2 to 24 weeks (27). In particular, the Cochrane review on schizophrenia by Jones et al. (28) defined standard CBT as lasting 16–24 weeks, whereas shorter interventions were categorized as brief CBT. Building on this framework, we further subdivided brief CBT into short-term (1–8 weeks) and medium-term (8–16 weeks), while interventions extending beyond 16 weeks were classified as long-term. This approach captures the variability of treatment durations across trials and is consistent with accepted standards for subgroup analyses (29).

### 2.5 Quality assessment

Based on the Cochrane Handbook for Systematic Reviews of Interventions V5.1.0, we used RevMan 5.4 software (Cochrane Collaboration, London, UK) to perform risk-of-bias assessment with the Cochrane Risk of Bias (RoB 1.0) tool. The methodological quality of each included RCT was assessed across seven domains: random sequence generation, allocation concealment, blinding of participants, blinding of outcome assessment, incomplete outcome data, selective reporting and other potential sources of bias. Each domain was judged as “low risk”, “high risk”, or “unclear risk” of bias. Furthermore, an overall quality grade was assigned according to the evaluation results: studies with all items rated as “low risk” were classified as Grade A; those with some items rated as “low risk” were classified as Grade B; and those with no items rated as “low risk” were classified as Grade C (30).

### 2.6 Data analysis

All analyses were performed using Stata18.0 software (StataCorp, College Station, TX, USA). Outcome measures were calculated using standardized mean differences (SMDs) represented by Hedges’g with corresponding 95% confidence intervals (CIs). The results of the meta-analysis are shown in the form of a forest plot of included studies, showing the first author, the year of publication, the individual effects and the overall effect with the 95% CI. Statistical heterogeneity across studies was quantified using the I² statistic, with I² < 50% indicating no significant heterogeneity. When the criteria were met, a fixed-effect model was applied; otherwise, a random-effects model was used (31). Forest plots were generated to display individual and pooled effect sizes, where square sizes represented study weights and diamond shapes indicated overall estimate. Sensitivity analyses were performed sequentially excluding individual studies to assess the stability of the pooled effect sizes. Moreover, studies were stratified according to the duration of CBT interventions, and effect sizes as well as within-subgroup variances were calculated accordingly. To address potential publication bias, funnel plots were visually inspected, and Egger’s regression test was applied, with a two-tailed p-value < 0.05 considered to indicate statistically significant publication bias. Finally, the GRADE framework was applied to assess evidence quality (32).

## 3 Results

### 3.1 Study description

The initial search provided a total of 9671 records. After removing duplicates, 8319 reports were screened based on the title and abstract, of which 28 full-text reports were assessed for inclusion. 14 reports were not eligible for inclusion in the review. Finally, 15 RCTs were included in this review (33–47) (**Figure 1**).

A total of 2135 elderly with AD were participated in the included studies, with intervention durations ranging from 5 weeks to 2 years. The characteristics of the included studies, including study (first author), year, country, sample size, gender distribution (M/F), mean age, intervention, duration, and outcomes, are summarized in **Table 2**.

**Table 2:** Characteristics of Included Studies.

Study	Year	Country	Sample size		Gender(m/f)	Mean age		Intervention		Duration	Outcomes
			EG	CG		EG	CG	EG	CG		
Davis (33)	2001	USA	20	20	16/21	68.7	72.6	Weekly 1-hour individual clinic sessions for 5 weeks and home-based attention exercises for 30 minutes per day, 6 days a week, over 4–5 weeks.	Unstructured conversation	5 weeks	MMSE, GDS, QoL
Burns (34)	2005	UK	20	20	21/19	73.9	77.7	Six weekly 50-minute sessions of psychodynamic interpersonal therapy conducted by trained therapists.	Standard care	6 weeks	MMSE, CSDD
Niu (35)	2010	China	16	16	25/7	80.6	79.1	Cognitive stimulation therapy, twice a week for 10 weeks, each session lasting 45 minutes,	Communications without cognitive training components	10 weeks	MMSE, NPI
Kurz (36)	2012	Germany	100	101	113/88	72.4	75	2 weekly 1-hour cognitive rehabilitation sessions structured into thematic modules over 12 weeks.	Usual site-specific medical management	12 weeks	MMSE, GDS, NPI, QoL
Bergamaschi (37)	2013	Italy	16	16	–	78.2	77.7	Five 1-month cycles of cognitive training (one cycle: 20 sessions, 2 h per day, 5 days a week) with a break of 4 weeks in between each cycle	Non-specific cognitive activity	20 weeks	MMSE, CSDD
Phung (38)	2013	Denmark	163	167	151/179	76.5	75.9	a multifaceted psychosocial intervention over 8–12 months, including at least 3 individual counseling sessions and 3 group education sessions	Follow-up support	12 months	MMSE, GDS, NPI, QoL
Quintana-Hernández (39)	2014	Spain	35 27 33	25	54/66	80.1	80.1	Three weekly 90-minute sessions over 96 weeks (totaling 288 sessions), receiving either mindfulness-based stress reduction (MBSR),progressive muscle relaxation (PMR), or cognitive stimulation therapy (CST)	Usual care	2 years	MMSE, GDS, NPI
Amieva (40)	2015	France	170 172 157	154	257/389	78.5 78.8 78.9	78.7	Weekly 90-minute sessions of cognitive training(CT), reminiscence therapy(RT), or cognitive rehabilitation(CR) for the first 3 months, followed by maintenance sessions every 6 weeks for 21 months.	Usual care	2 years	NPI, QoL
Koivisto (41)	2015	Finland	84	152	115/121	75.5	75.8	Four rehabilitation courses over 2 years, each course lasting 4 days, combining individual counseling, education, and group support.	No treatment	2 years	MMSE. NPI, QoL
Tsantali (42)	2017	Greece	17 17	21	–	73.4 73.3	74.2	Cognitive Training(CT) or Cognitive Stimulation(CS), three times per week with each session lasting 90 minutes for 16 weeks	No intervention	4 months	MMSE
Trebbastoni (44)	2018	Italy	45	85	52/78	74.3	76.0	Cognitive training session lasting approximately 70 minutes, twice a week for six months	Usual treatment	6 months	MMSE
Cavallo (43)	2018	Italy	40	40	–	–	–	Computerized cognitive training three times per week for 12 weeks	Computer-based leisure activities	12 weeks	MMSE
Li (45)	2019	China	43	42	47/38	83.2	83.5	Reminiscence therapy twice a week for 12 weeks, with each session lasting 30–45 minutes	Usual treatment	12 weeks	CSDD, NPI
Lök (46)	2019	Turkey	30	30	26/34	–	–	Weekly 60-minute reminiscence therapy sessions for 8 weeks	No intervention	8 weeks	MMSE, CSDD, QoL
Forstmeier (47)	2025	Germany	20	21	14/26	74.9	76.2	Cognitive behavioral therapy consisting of approximately 25 weekly sessions tailored, each session lasting about 1 hour	Usual treatment	6 months	CSDD, GDS, NPI, QoL

A total of 15 RCTs published between 2001 and 2025 were included, conducted across 11 countries. EG, Experiment Group; CG, Control Group; M/F, Male/Female; -, Missing data; MMSE, Mini-Mental State Examination; CSDD, Cornell Scale for Depression in Dementia; GDS, Geriatric Depression Scale; NPI, Neuropsychiatric Inventory; QoL, quality of life.

### 3.2 Quality assessment of the included studies

**Figure 2:**
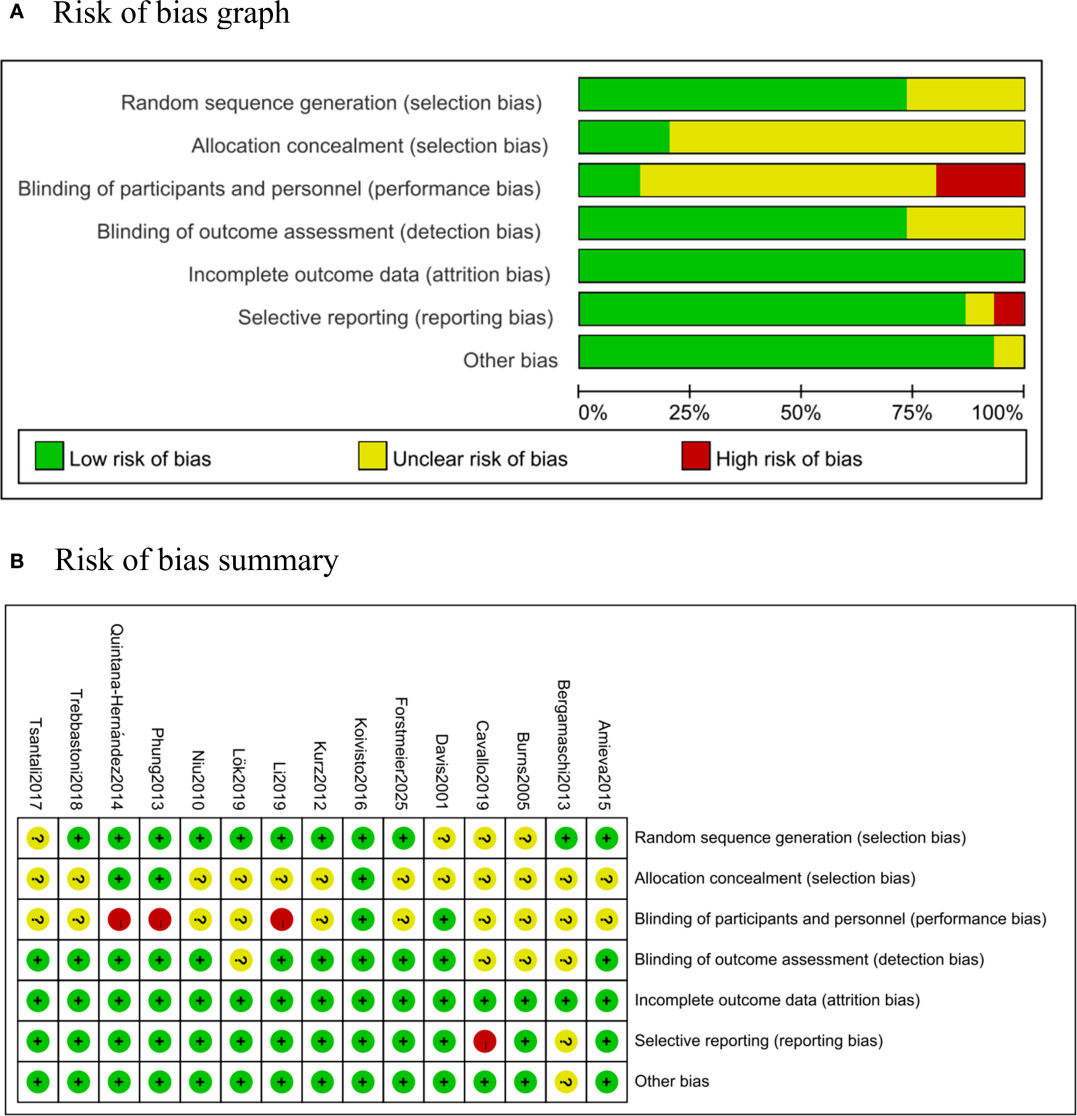
Risk of bias. **(A)** Risk of bias graph: percentages of each risk of bias item across all studies, as judged by the review authors. **(B)** Risk of bias summary: assessment of each risk of bias item in every included study, as evaluated by the review authors.

The methodological quality of included studies was evaluated using the Cochrane Collaboration Recommendations assessment tools RevMan 5.4. (**Figure 2**). One trial was graded as A (41), and 14 were graded as B (37–40, 42–47). Randomization sequences were adequately described in 11 studies, while allocation concealment was clearly implemented in 3 studies. 2 studies reported blinding of both participants and assessors. All included trials reported their primary outcome indicators.

### 3.3 Meta-analysis of AD outcomes

#### 3.3.1 Global cognition

**Figure 3:**
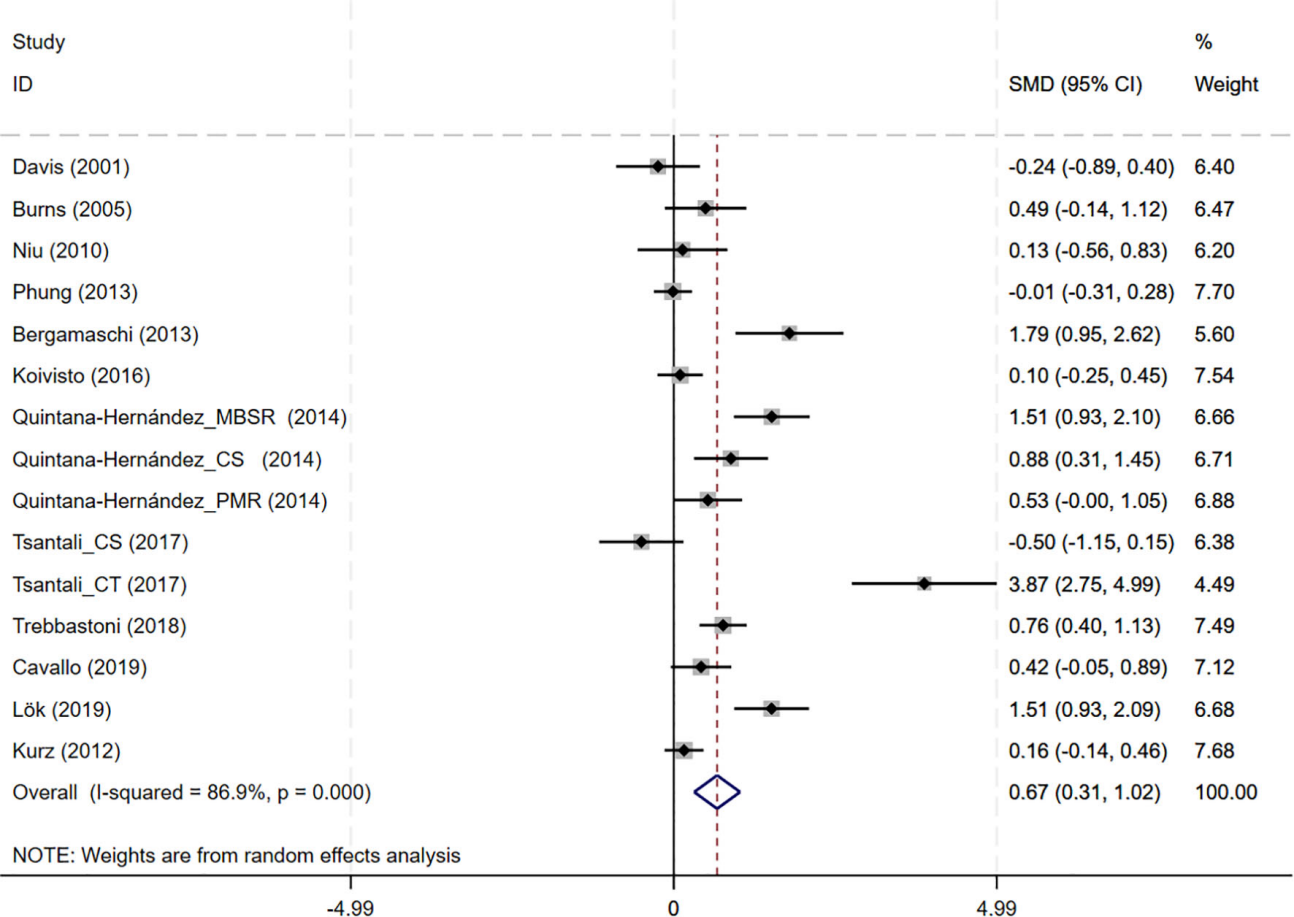
Forest plot for cognitive behavioral therapy on global cognition (MMSE). MMSE, Mini-Mental State Examination.

CBT interventions showed a significant improvement in MMSE outcomes compared to the controls (12 studies, SMD = 0.668, 95%CI:0.314-1.023, p<0.001; I^2^ = 86.9%) (**Figure 3**). The funnel plot appeared largely symmetrical, suggesting no obvious publication bias (**Supplementary Figure S2A**). However, Egger’s test indicated potential publication bias (p = 0.025), implying that small-study effects might have influenced the results.

#### 3.3.2 Depression severity (CSDD, GDS)

**Figure 4:**
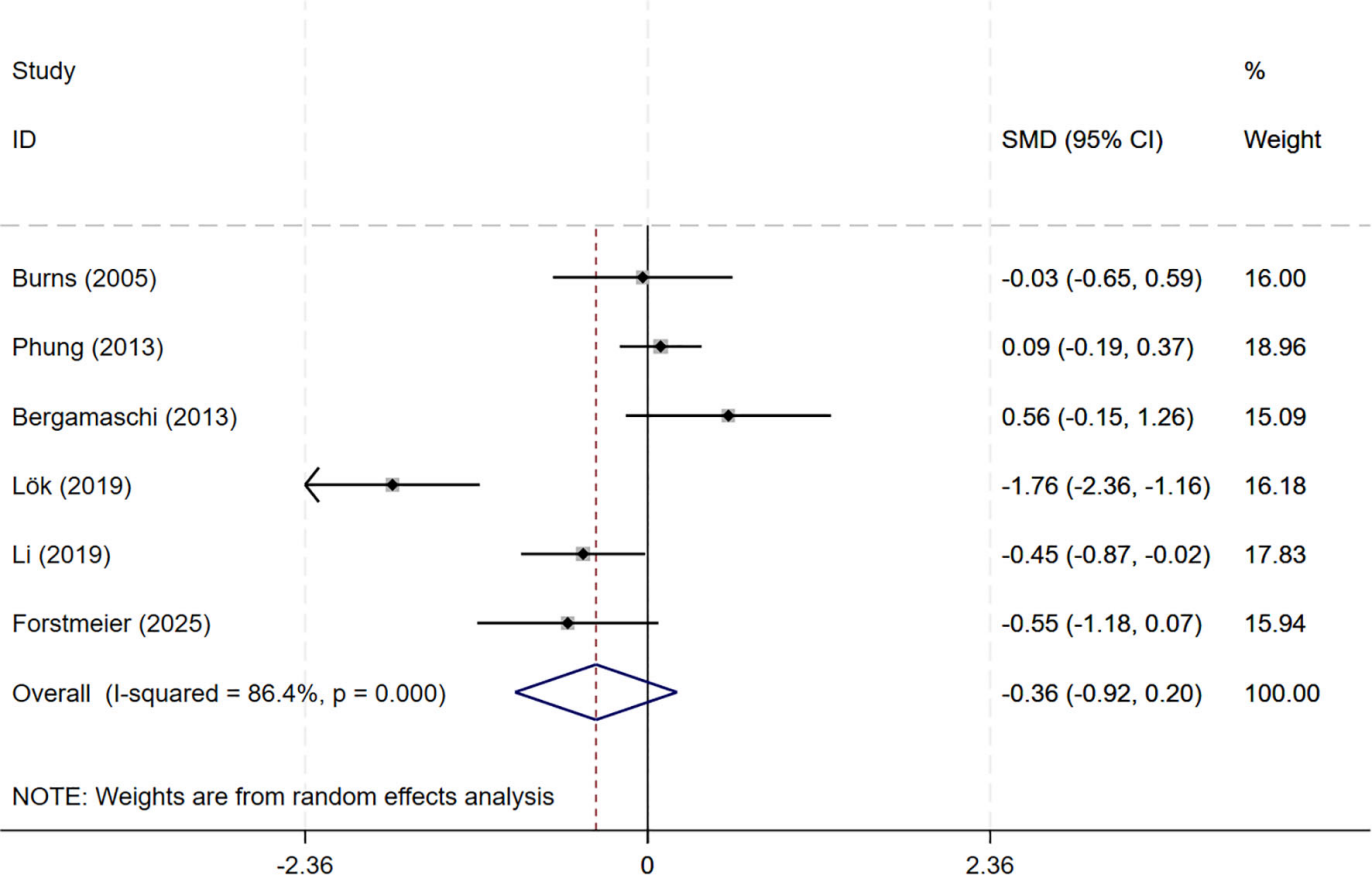
Forest plot for cognitive behavioral therapy on depression severity (CSDD). CSDD, Cornell Scale for Depression in Dementia.

**Figure 5:**
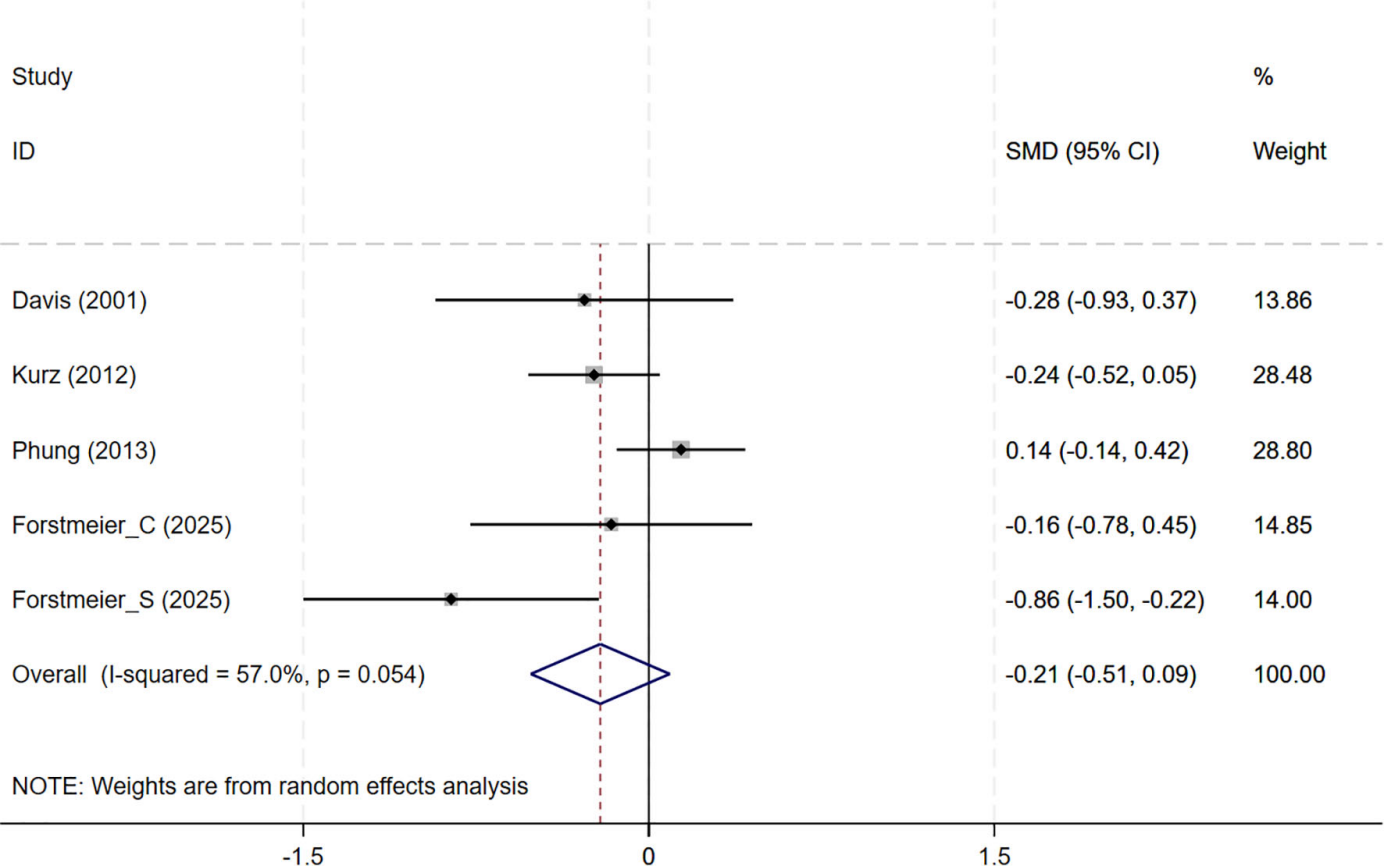
Forest plot for cognitive behavioral therapy on depression severity (GDS). GDS, Geriatric Depression Scale.

In total, 8 studies involving depression dimension were included, and meta-analysis show no evidence of depression mood improvement with CBT interventions (CSDD: 6 studies, SMD= -0.357, 95%CI: -0.915 to 0.202, p=0.211; I^2^ = 86.4%, GDS: 4 studies, SMD=-0.211, 95%CI: -0.514 to 0.091, p=0.170; I^2^ = 57.0%) (**Figures 4**, **5**). Visual inspection showed that the funnel plot for GDS was asymmetric (**Supplementary Figure S2C**), whereas the other plot appeared largely symmetrical (**Supplementary Figure S2B**). Egger’s tests did not detect significant publication bias (CSDD: p = 0.478; GDS: p = 0.271).

#### 3.3.3 Behavioral and psychological symptoms (NPI)

**Figure 6:**
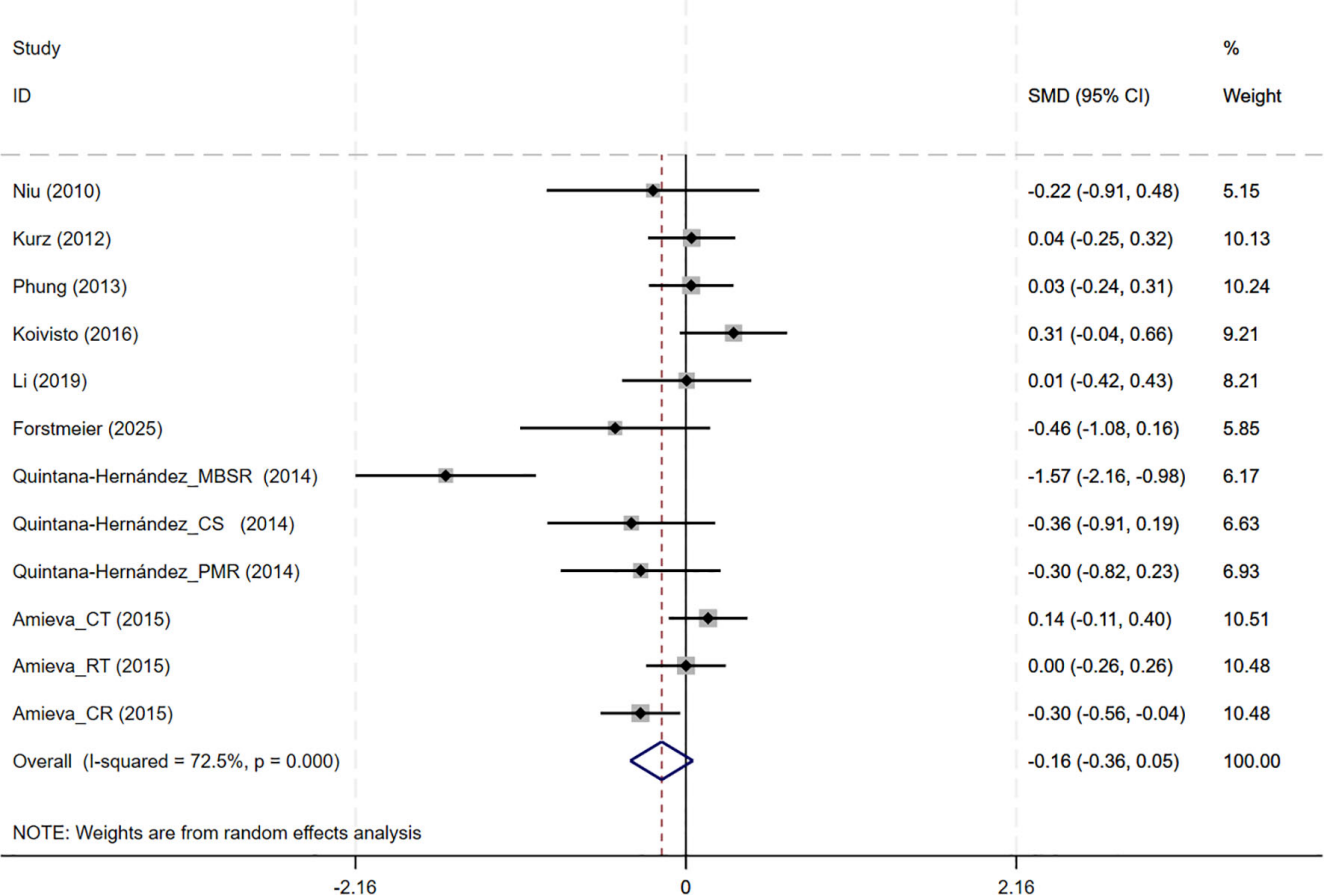
Forest plot for cognitive behavioral therapy on behavioral and psychological symptoms (NPI). NPI, Neuropsychiatric Inventory.

In total, 8 studies involving NPI outcomes were included, and meta-analysis show no evidence of behavioral and psychological improvement with CBT interventions(8 studies, SMD=-0.159, 95%CI: -0.364 to 0.046, p=0.129; I^2^ = 72.5%) (**Figure 6**). Neither the funnel plot (**Supplementary Figure S2D**) nor Egger’s test (p=0.071) indicated the presence of publication bias.

#### 3.3.4 Subjective wellbeing (QoL)

**Figure 7:**
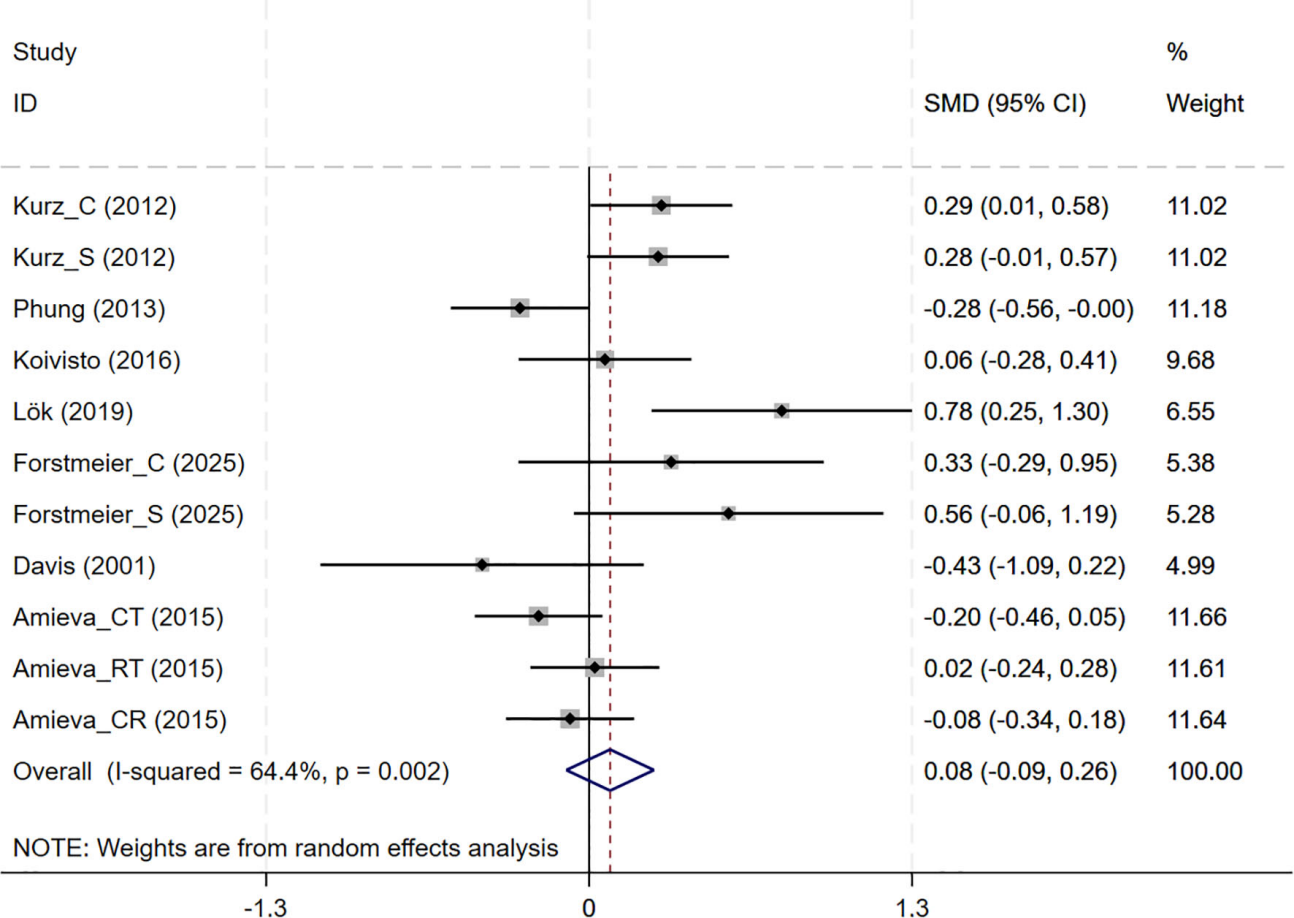
Forest plot for cognitive behavioral therapy on Subjective Wellbeing (QoL). QoL, Quality of Life.

In total, 7 studies involving QoL outcomes were included. Among them, the studies by Kurz and Forstmeier (36, 47) assessed both self-rated and caregiver-rated QoL scores. And CBT interventions showed no differential effects across subjective wellbeing (7 studies, SMD = 0.085, 95%CI: -0.092 to 0.262, p= 0.348; I^2^ = 64.4%) (**Figure 7**). The results of the funnel plot (**Supplementary Figure S2E**), along with Egger’s test (p=0.220), revealed no significant signs of publication bias.

### 3.4 Sensitivity analyses

Leave-one-out Sensitivity analyses were conducted between the CBT intervention group and the controls for MMSE, GDS, CSDD, NPI and QoL. The results indicated that no single study had a disproportionate influence on the overall estimate, as the pooled effect sizes remained consistent and within similar confidence intervals when each study was sequentially excluded (**Supplementary Figure S3**). This suggests that the meta-analytic findings are stable and not driven by any individual study.

### 3.5 Subgroup analyses

**Figure 8:**
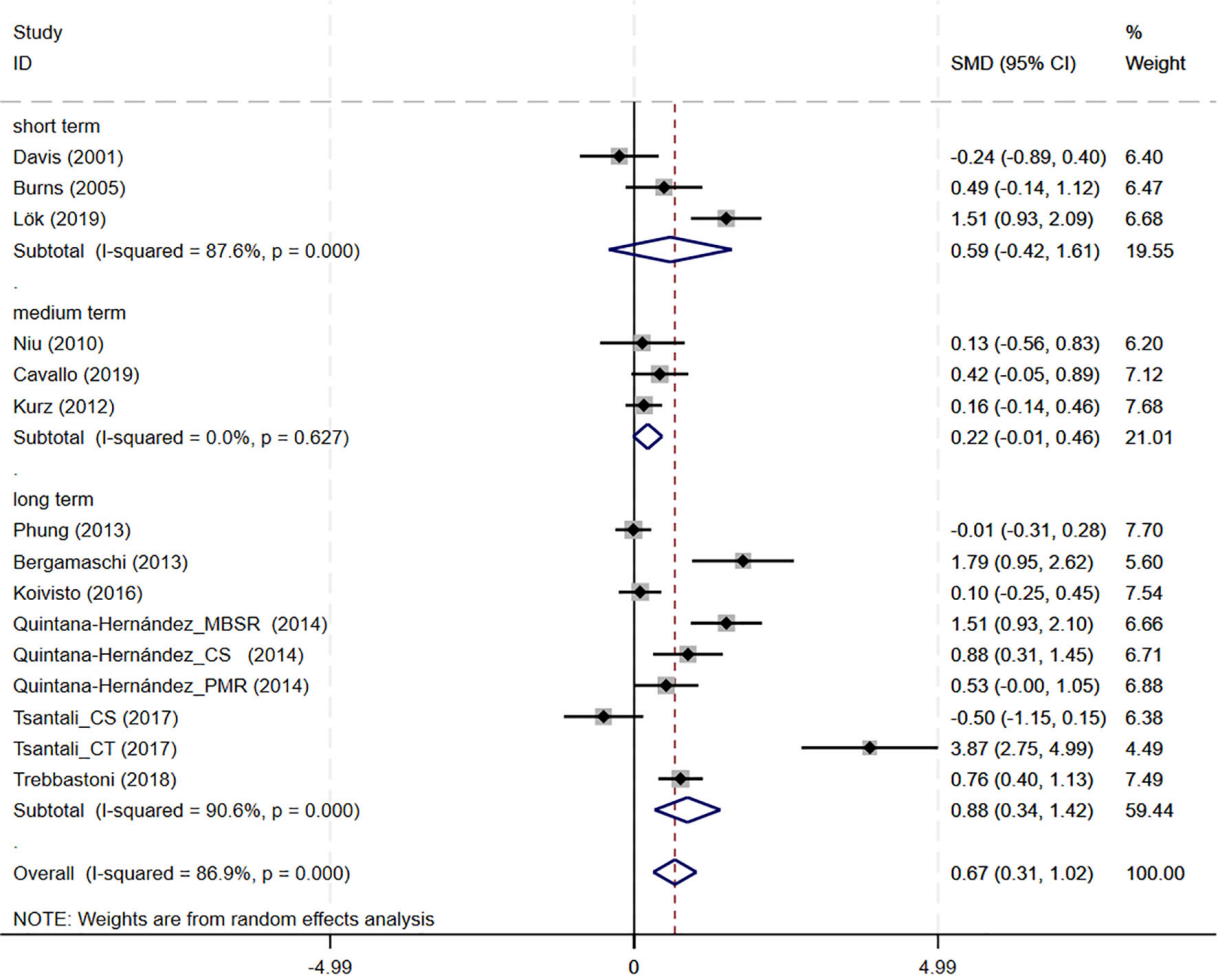
Subgroup analysis of MMSE according to cognitive behavioral therapy duration. MMSE, Mini-Mental State Examination. short-term: 1–8 weeks; medium term: 8–16 weeks; long term: >16 weeks.

**Figure 9:**
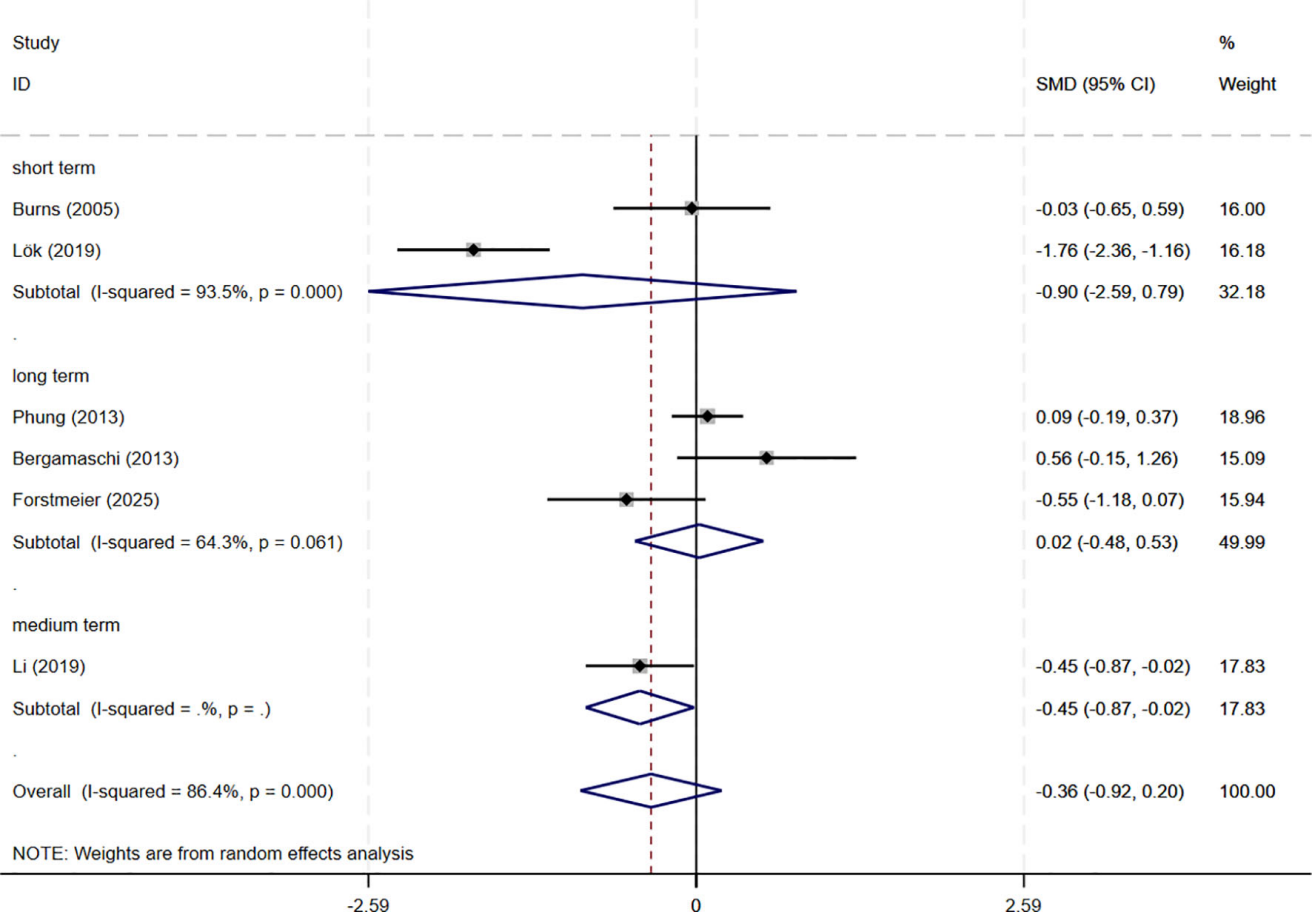
Subgroup analysis of CSDD according to cognitive behavioral therapy duration. CSDD, Cornell Scale for Depression in Dementia; short-term: 1–8 weeks; medium term: 8–16 weeks; long term: >16 weeks.

**Figure 10:**
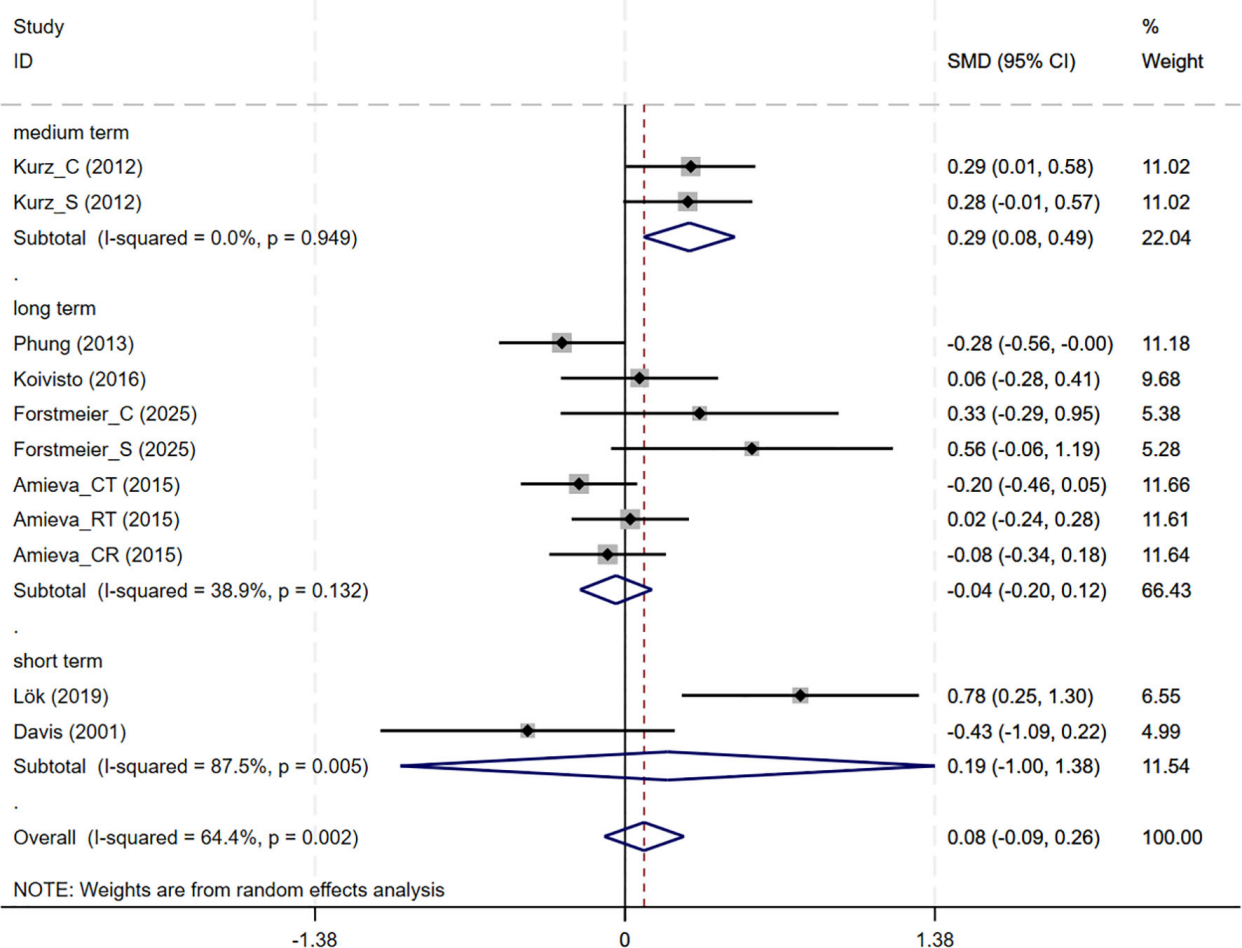
Subgroup analysis of QoL according to cognitive behavioral therapy duration. QoL, Quality of Life; short-term: 1–8 weeks; medium term: 8–16 weeks; long term: >16 weeks.

Subgroup analyses were conducted to explore the impact of intervention duration on treatment outcomes. Results indicated that long-term CBT was associated with greater improvements in global cognitive function, as assessed by the MMSE, compared to short- and medium-term interventions (SMD = 0.876, 95%CI: 0.337 to 1.415) (**Figure 8**). Studies evaluating depressive symptoms using CSDD showed that medium-term CBT produced notable improvements in mood-related outcomes (SMD=-0.445, 95%CI: -0.874 to -0.017) (**Figure 9**). Furthermore, studies assessing subjective well-being via QoL measures also demonstrated that medium-term CBT elicited favorable effects on emotional well-being (SMD = 0.285, 95%CI: 0.083 to 0.488) (**Figure 10**). Subgroup analyses for other outcomes showed no significant differences (**Supplementary Figure S4**).

### 3.6 Publication bias and evidence quality

Publication bias was assessed using both funnel plots and Egger’s test. Funnel plots suggested potential publication bias for the GDS outcome, whereas no obvious bias was observed for the other outcomes. Egger’s test indicated significant publication bias for MMSE, suggesting that the results for MMSE should be interpreted with caution.

The quality of evidence was evaluated using the GRADE approach. Overall, the evidence ranged from low to moderate, mainly due to heterogeneity, risk of bias, imprecision, and some concerns regarding publication bias. (see **Supplementary Table S5** for details).

## 4 Discussion

Given the established efficacy of CBT (cognitive behavioral therapy) in treating mood disorders such as depression and anxiety (10), its techniques have been progressively adapted to address the specific needs of AD patients experiencing cognitive decline. This systematic review and meta-analysis assessed the effects of CBT on global cognition, depression severity, neuropsychiatric Symptoms, and life satisfaction in elderly patients with AD. The pooled results suggest that CBT may be an effective intervention to improve cognitive decline in AD. Furthermore, subgroup analyses indicated that CBT cycles of 8–16 weeks were beneficial in improving depression and quality of life in AD patients. These findings provide important evidence for the potential of CBT to address multiple domains of impairment in AD and warrant further exploration in future clinical applications.

Regarding cognitive outcomes, CBT intervention groups showed significant cognitive improvement compared with the control group after treatment, and subgroup analyses showed that long-term CBT was particularly beneficial. When the observed SMD (0.67) is translated into MMSE points using the pooled SD, it corresponds to an estimated 2.87-point improvement, suggesting clinically meaningful benefits of CBT for cognitive function in AD patients (see **Supplementary Table S6** for details). These results are consistent with previous findings from other psychosocial interventions. An umbrella review found that cognitive interventions consistently improved MMSE scores in dementia (8). Likewise, an evidence synthesis noted that enhanced brain plasticity underpins such improvements and that cognitive training showed a small but significant global cognitive gain (48). These findings are generally in line with observation of improved MMSE scores post-CBT intervention. Neuroimaging evidence shows that such training boots functional connectivity in learning and memory networks even in AD patients. For example, Behfar and colleagues found that cognitive stimulation therapy can lead to a significant improvement in MMSE scores and an increase in resting-state connectivity in memory region (49). These results prove that neuroplasticity can be harnessed to alleviate cognitive impairment in AD. In other words, repeated cognitive exercises may strengthen connections at the synapse or recruit compensatory networks, thus enhancing cognitive reserve. This plasticity-based mechanism aligns with our findings of cognitive benefit after CBT interventions. Beyond cognition, neuropsychiatric symptoms and quality of life are also important domains to consider when evaluating the impact of CBT in AD.

Meta-analyses and systematic reviews also indicate that CBT-based treatments confer modest mood and improve quality of life. A Cochrane review found that CBT which includes behavioral activation and problem-solving therapy probably slightly reduces depressive symptoms in dementia (SMD =–0.23) and modestly improves quality of life (SMD = 0.31) (18). A recent systematic review similarly found that CBT reduces depression in AD patients, although certainty is limited due to heterogeneity (50). These findings are in agreement with results from our subgroup analyses which showed medium-term CBT lowered CSDD scores and improved QoL compared to long and short-term CBT. Interventions based on CBT work by identifying and modifying maladaptive thoughts and behaviors. By challenging negative automatic beliefs and teaching problem-solving or activity-planning skills, CBT reduces depressive effect and increases engagement in adaptive behaviors. In dementia care, this means helping patients reinterpret stressors and re-engage in something more meaningful. As a result, it yields small improvements in mood and daily activities. This mechanism accounts for reductions in CSDD scores and improvement QoL in our meta-analysis of CBT.

Despite these encouraging findings, several limitations should be acknowledged when interpreting the results of this meta-analysis. First, the number of studies was small (n=15), which reduces statistical power and the robustness of conclusions. The issues include difficulty in recruitment, treatment fidelity, and compliance. In addition, the presence of several small-sample trials raises concern for small-study effects: while the MMSE funnel plot did not display clear asymmetry on visual inspection, Egger’s test indicated a statistically significant bias, suggesting that smaller studies with positive results may be overrepresented. Consequently, the magnitude of the positive effects observed could be overestimated and our results should be interpreted cautiously. Second, significant heterogeneity was observed in among trials (MMSE: I²=86.9%). This variability may stem from difference in study populations (age, gender distribution, dementia severity), intervention protocols (frequency, delivery format), control conditions, methodological quality (inadequate allocation concealment, limited blinding) and sample size variation. To better understand this, we summarized potential sources across studies (**Supplementary Table S7**). Besides, the use of MMSE, which is a relatively crude screening tool, may have further amplified inconsistencies and contributed to potential publication bias. Third, while subgroup analyses suggested improvements in depressive symptoms, global neuropsychiatric outcomes (NPI) did not show significant changes, suggesting that CBT may have limited effects on broader neuropsychiatric symptoms in AD. One possible explanation is that CBT primarily targets mood-related symptoms such as depression and anxiety. By contrast, other domains captured by the NPI, such as delusions, hallucinations, and disinhibition, are more closely linked to neurodegenerative processes and may therefore be less responsive to psychological interventions. In addition, limitations of the NPI itself should be considered: the tool relies on caregiver reports, may lack sensitivity to subtle improvements, and its composite scoring may dilute specific benefits of CBT on mood-related symptoms. Finally, most trials failed to adequately address allocation concealment, and only two implemented participant blinding. The inability to blind participants is a common challenge in psychological intervention studies (51). Because psychological interventions are difficult to disguise, participants often recognize their group allocation, which increases the risk of expectancy bias. These challenges in participant blinding underscore the broader importance of rigorous trial design, including proper allocation concealment. In our study, both participant blinding and allocation concealment were difficult to achieve. This is particularly concerning subjective outcomes such as depression and quality of life, where empirical evidence suggests that lack of blinding and inadequate allocation concealment can lead to exaggerated intervention effect estimates (52). Collectively, these factors led to downgrading the certainty of evidence; according to the GRADE framework, the certainty of evidence ranged from low to moderate across different outcomes, and the results should therefore be interpreted with caution.

To our knowledge, systematic evaluations of CBT in AD through meta-analyses are relatively few, and subgroup analyses considering treatment duration have seldom been explored. By including a comprehensive literature search and strict selection of RCTs, this study provides preliminary evidence on the effects of CBT on cognitive and neuropsychiatric outcomes. With limited drugs available to postpone cognitive decline and manage neuropsychiatric symptoms in AD, these findings indicate that CBT may be a useful, low-risk, and scalable psychological treatment for AD. Importantly, the blinding of outcome assessment was reasonably maintained. In addition, both attrition bias and selective reporting appeared limited. Despite the presence of significant statistical heterogeneity, sensitivity analyses demonstrated that the findings for all outcomes remained stable, which collectively supports the reliability of these results. It is reasonable to consider that incorporating structured CBT programs into standard care may provide clinicians with a practical approach to enhance patients’ cognitive engagement and emotional well-being. Given remaining limitations in the evidence base, future research should prioritize larger, methodologically rigorous trials with extended follow-up periods. These studies are needed to more conclusively determine the optimal type and duration of CBT interventions for improving or maintaining cognitive function, emotional well-being, and quality of life in patients with AD.

## 5 Conclusions

Early cognitive decline is the hallmark of AD, which imposes a substantial emotional and physical burden on patients and caregivers. This meta-analysis provides preliminary evidence that CBT may be effective in slowing cognitive decline in patients with AD. Subgroup analysis suggests that CBT interventions lasting 8–16 weeks may also help improve depressive symptoms and quality of life. However, the findings should be interpreted with caution due to the limited number of studies, small sample sizes, and considerable heterogeneity in population, such as age and gender distribution. Additionally, the potential for publication bias and unclear blinding procedures in several trials may have influenced the outcomes. Despite these limitations, CBT appears to be a promising, low-risk non-pharmacological approach for managing cognitive and emotional symptoms in AD. Future high-quality, large-scale randomized controlled trials using standardized CBT protocols are needed to confirm these findings and inform clinical application.
