# Supplementary material for: Effects of cognitive behavioral therapy on cognition, neuropsychiatric symptoms, and quality of life in Alzheimer’s disease: a meta-analysis
Source: Front Psychiatry. 2025 Sep 29;16:1648225. doi: 10.3389/fpsyt.2025.1648225 (PMC12515942; doi:10.3389/fpsyt.2025.1648225)
Supplement: Supplementary file 1 [file DataSheet1.pdf]

SUPPLEMENTARY MATERIAL

Table of Contents

Supplementary Method S1. Database search strategy
Supplementary Figure S2. Funnel plots
Supplementary Figure S2a. Funnel plots for MMSE
Supplementary Figure S2b. Funnel plots for CSDD
Supplementary Figure S2c. Funnel plots for GDS
Supplementary Figure S2d. Funnel plots for NPI
Supplementary Figure S2e. Funnel plots for QoL
Supplementary Figure S3. Sensitivity analyses
Supplementary Figure S3a. Sensitivity analysis for MMSE
Supplementary Figure S3b. Sensitivity analysis for CSDD
Supplementary Figure S3c. Sensitivity analysis for GDS
Supplementary Figure S3d. Sensitivity analysis for NPI
Supplementary Figure S3e. Sensitivity analysis for QoL
Supplementary Figure S4. Subgroup analyses
Supplementary Figure S4a. Subgroup analysis for GDS
Supplementary Figure S4b. Subgroup analysis for NPI
Supplementary Table S5. GRADE evidence summary
Supplementary Table S6. Calculation of pooled SD for MMSE
Supplementary Table S7. Potential sources of heterogeneity

Supplementary Method S1. Database search strategy

We searched PubMed, Embase, Cochrane Library, and Web of Science up to March 27, 2025. For all databases, search strategies combined subject headings with relevant free-text terms related to Alzheimer's disease and cognitive behavioral therapy.

1.1 PubMed

No.	Search Details	Results
#1	"alzheimer disease"[MeSH Terms]	132,076
#2	"alzheimer disease"[Title/Abstract] OR "alzheimer syndrome"[Title/Abstract] OR "alzheimer type dementia atd"[Title/Abstract] OR "alzheimer type dementia atd"[Title/Abstract] OR "alzheimer diseases"[Title/Abstract] OR "alzheimers diseases"[Title/Abstract] OR "alzheimer dementia"[Title/Abstract] OR "alzheimer dementias"[Title/Abstract] OR "senile dementia"[Title/Abstract] OR "alzheimer type dementia"[Title/Abstract] OR "alzheimer type senile dementia"[Title/Abstract] OR "primary senile degenerative dementia"[Title/Abstract] OR "alzheimer sclerosis"[Title/Abstract] OR "presenile dementia"[Title/Abstract] OR "acute confusional senile dementia"[Title/Abstract] OR "early onset alzheimer disease"[Title/Abstract] OR "presenile alzheimer dementia"[Title/Abstract] OR "late onset alzheimer disease"[Title/Abstract] OR "familial alzheimer disease fad"[Title/Abstract]	28,252
#3	"alzheimer disease"[MeSH Terms] OR "alzheimer disease"[Title/Abstract] OR "alzheimer syndrome"[Title/Abstract] OR "alzheimer type dementia atd"[Title/Abstract] OR "alzheimer type dementia atd"[Title/Abstract] OR "alzheimer diseases"[Title/Abstract] OR "alzheimers diseases"[Title/Abstract] OR "alzheimer dementia"[Title/Abstract] OR "alzheimer dementias"[Title/Abstract] OR "senile dementia"[Title/Abstract] OR "alzheimer type dementia"[Title/Abstract] OR "alzheimer type senile dementia"[Title/Abstract] OR "primary senile degenerative dementia"[Title/Abstract] OR "alzheimer sclerosis"[Title/Abstract] OR "presenile dementia"[Title/Abstract] OR "acute confusional senile dementia"[Title/Abstract] OR "early onset alzheimer disease"[Title/Abstract] OR "presenile alzheimer dementia"[Title/Abstract] OR "late onset alzheimer disease"[Title/Abstract] OR "familial alzheimer disease fad"[Title/Abstract]	142,929
#4	"cognitive behavioral therapy"[MeSH Terms]	40,598
#5	"cognitive behavioral therapy"[Title/Abstract] OR "cognitive behavioral therapies"[Title/Abstract] OR "cognition therapy"[Title/Abstract] OR (("Cognition"[MeSH Terms] OR "Cognition"[All Fields] OR "cognitions"[All Fields] OR "Cognitive"[All Fields] OR "cognitively"[All Fields] OR "cognitives"[All Fields]) AND "Therapies"[Title/Abstract]) OR	34,779

	"cognitive behavior therapies"[Title/Abstract] OR "cognitive behavior therapy"[Title/Abstract] OR "cognitive psychotherapy"[Title/Abstract] OR "cognitive psychotherapies"[Title/Abstract] OR "cognitive therapies"[Title/Abstract] OR "cognitive behaviour therapy"[Title/Abstract] OR "cognitive behaviour therapies"[Title/Abstract]	
#6	"cognitive behavioral therapy"[MeSH Terms] OR ("cognitive behavioral therapy"[Title/Abstract] OR "cognitive behavioral therapies"[Title/Abstract] OR "cognition therapy"[Title/Abstract] OR (("Cognition"[MeSH Terms] OR "Cognition"[All Fields] OR "cognitions"[All Fields] OR "Cognitive"[All Fields] OR "cognitively"[All Fields] OR "cognitives"[All Fields]) AND "Therapies"[Title/Abstract]) OR "cognitive behavior therapies"[Title/Abstract] OR "cognitive behavior therapy"[Title/Abstract] OR "cognitive psychotherapy"[Title/Abstract] OR "cognitive psychotherapies"[Title/Abstract] OR "cognitive therapies"[Title/Abstract] OR "cognitive behaviour therapy"[Title/Abstract] OR "cognitive behaviour therapies"[Title/Abstract] OR "cognitive therapy"[Title/Abstract])	62,839
#7	("alzheimer disease"[MeSH Terms] OR ("alzheimer disease"[Title/Abstract] OR "alzheimer syndrome"[Title/Abstract] OR "alzheimer type dementia atd"[Title/Abstract] OR "alzheimer type dementia atd"[Title/Abstract] OR "alzheimer diseases"[Title/Abstract] OR "alzheimers diseases"[Title/Abstract] OR "alzheimer dementia"[Title/Abstract] OR "alzheimer dementias"[Title/Abstract] OR "senile dementia"[Title/Abstract] OR "alzheimer type dementia"[Title/Abstract] OR "alzheimer type senile dementia"[Title/Abstract] OR "primary senile degenerative dementia"[Title/Abstract] OR "alzheimer sclerosis"[Title/Abstract] OR "presenile dementia"[Title/Abstract] OR "acute confusional senile dementia"[Title/Abstract] OR "early onset alzheimer disease"[Title/Abstract] OR "presenile alzheimer dementia"[Title/Abstract] OR "late onset alzheimer disease"[Title/Abstract] OR "familial alzheimer disease fad"[Title/Abstract])) AND ("cognitive behavioral therapy"[MeSH Terms] OR ("cognitive behavioral therapy"[Title/Abstract] OR "cognitive behavioral therapies"[Title/Abstract] OR "cognition therapy"[Title/Abstract] OR (("Cognition"[MeSH Terms] OR "Cognition"[All Fields] OR "cognitions"[All Fields] OR "Cognitive"[All Fields] OR "cognitively"[All Fields] OR "cognitives"[All Fields]) AND "Therapies"[Title/Abstract]) OR "cognitive behavior therapies"[Title/Abstract] OR "cognitive behavior therapy"[Title/Abstract] OR "cognitive psychotherapy"[Title/Abstract] OR "cognitive psychotherapies"[Title/Abstract] OR "cognitive therapies"[Title/Abstract] OR "cognitive behaviour therapy"[Title/Abstract] OR "cognitive behaviour therapies"[Title/Abstract] OR "cognitive therapy"[Title/Abstract]))	2,162

Search Date: 27-Mar-2025

1.2 Embase

No.	Search Details	Results
#1	'alzheimer disease'/exp	276022
#2	'alzheimer disease':ab,ti	26547
#3	'alzheimer syndrome':ab,ti	29
#4	'alzheimer-type dementia (atd)':ab,ti	127
#5	'alzheimer type dementia (atd)':ab,ti	127
#6	'alzheimer diseases':ab,ti	288
#7	'alzheimers diseases':ab,ti	14
#8	'alzheimer dementia':ab,ti	1102
#9	'alzheimer dementias':ab,ti	79
#10	'senile dementia':ab,ti	3863
#11	'alzheimer type dementia':ab,ti	927
#12	'alzheimer type senile dementia':ab,ti	24
#13	'primary senile degenerative dementia':ab,ti	0
#14	'alzheimer sclerosis':ab,ti	0
#15	'presenile dementia':ab,ti	759
#16	'acute confusional senile dementia':ab,ti	0
#17	'early onset alzheimer disease':ab,ti	242
#18	'presenile alzheimer dementia':ab,ti	3
#19	'late onset alzheimer disease':ab,ti	542
#20	'familial alzheimer disease (fad)':ab,ti	99
#21	'familial alzheimer diseases (fad)':ab,ti	0
#22	#1 OR #2 OR #3 OR #4 OR #5 OR #6 OR #7 OR #8 OR #9 OR #10 OR #11 OR #12 OR #13 OR #14 OR #15 OR #16 OR #17 OR #18 OR #19 OR #20 OR #21	282498
#23	'cognitive behavioral therapy'/exp	36035
#24	'cognitive behavioral therapy':ab,ti	18959
#25	'cognitive behavioral therapies':ab,ti	736
#26	'cognition therapy':ab,ti	11
#27	'cognition therapies':ab,ti	3
#28	'cognitive behavior therapies':ab,ti	79
#29	'cognitive behavior therapy':ab,ti	3838
#30	'cognitive psychotherapy':ab,ti	238
#31	'cognitive psychotherapies':ab,ti	279
#32	'cognitive therapies':ab,ti	565
#33	'cognitive therapy':ab,ti	5348
#34	'cognitive behaviour therapies':ab,ti	56
#35	'cognitive behavioral therapy':ab,ti	18959
#36	#23 OR #24 OR #25 OR #26 OR #27 OR #28 OR #29 OR #30 OR #31 OR #32 OR #33 OR #34 OR #35	52346
#37	#22 AND #36	395

Search Date: 27-Mar-2025

1.3 Cohrance library

No.	Search Details	Results
#1	MeSH descriptor: [Alzheimer Disease] explode all trees	5516
#2	(Alzheimer Disease):ti,ab,kw OR (Alzheimer Syndrome):ti,ab,kw OR (Alzheimer-Type Dementia (ATD)):ti,ab,kw OR (Alzheimer Type Dementia (ATD)):ti,ab,kw OR (Alzheimer Diseases):ti,ab,kw	14294
#3	(Alzheimers Diseases):ti,ab,kw OR (Alzheimer Dementia):ti,ab,kw OR (Alzheimer Dementias):ti,ab,kw OR (Senile Dementia):ti,ab,kw OR (Alzheimer Type Dementia):ti,ab,kw	7008
#4	(Alzheimer Type Senile Dementia):ti,ab,kw OR (Primary Senile Degenerative Dementia):ti,ab,kw OR (Alzheimer Sclerosis):ti,ab,kw OR (Presenile Dementia):ti,ab,kw OR (Acute Confusional Senile Dementia):ti,ab,kw	301
#5	(Early Onset Alzheimer Disease):ti,ab,kw OR (Presenile Alzheimer Dementia):ti,ab,kw OR (Late Onset Alzheimer Disease):ti,ab,kw OR (Familial Alzheimer Disease (FAD)):ti,ab,kw OR (Familial Alzheimer Diseases (FAD)):ti,ab,kw	325
#6	#1 or #2 or #3 or #4 or #5	14910
#7	MeSH descriptor: [Cognitive Behavioral Therapy] explode all trees	14291
#8	(Cognitive Behavioral Therapy):ti,ab,kw OR (Cognitive Behavioral Therapies):ti,ab,kw OR (Cognition Therapy):ti,ab,kw OR (Cognition Therapies):ti,ab,kw OR (Cognitive Behavior Therapies):ti,ab,kw	42477
#9	(Cognitive Behavior Therapy):ti,ab,kw OR (Cognitive Psychotherapy):ti,ab,kw OR (Cognitive Psychotherapies):ti,ab,kw OR (Cognitive Therapies):ti,ab,kw OR (Cognitive Behaviour Therapy):ti,ab,kw	19214
#10	(Cognitive Behaviour Therapies):ti,ab,kw OR (Cognitive Therapy):ti,ab,kw	54500
#11	#7 or #8 or #9 or #10	61041
#12	#6 and #11	4120

Search Date: 27-Mar-2025

1.4 Web of science

No.	Search Query	Results
#1	TS=(Alzheimer Disease) OR TS=(Alzheimer Syndrome) OR TS=(Alzheimer-Type Dementia (ATD)) OR TS=(Alzheimer Type Dementia (ATD)) OR TS=(Alzheimer Diseases) OR TS=(Alzheimers Diseases) OR TS=(Alzheimer Dementia) OR TS=(Alzheimer Dementias) OR TS=(Senile Dementia) OR TS=(Alzheimer Type Dementia) OR TS=(Alzheimer Type Senile Dementia) OR TS=(Primary Senile Degenerative Dementia) OR TS=(Alzheimer Sclerosis) OR TS=(Presenile Dementia) OR TS=(Acute Confusional Senile Dementia) OR TS=(Early Onset Alzheimer Disease) OR TS=(Presenile Alzheimer Dementia) OR	306375

	TS=(Late Onset Alzheimer Disease) OR TS=(Familial Alzheimer Disease (FAD)) OR TS=(Familial Alzheimer Diseases (FAD))	
#2	TS=(Cognitive Behavioral Therapy) OR TS=(Cognitive Behavioral Therapies) OR TS=(Cognition Therapy) OR TS=(Cognition Therapies) OR TS=(Cognitive Behavior Therapies) OR TS=(Cognitive Behavior Therapy) OR TS=(Cognitive Psychotherapy) OR TS=(Cognitive Psychotherapies) OR TS=(Cognitive Therapies) OR TS=(Cognitive Behaviour Therapy) OR TS=(Cognitive Behaviour Therapies) OR TS=(Cognitive Therapy)	115293
#3	#2 AND #1	12123
#4	TS=(randomized controlled trial) OR TS=(randomized) OR TS=(placebo) OR TS=(random) OR TS=(double-blind)	2163792
#5	#4 AND #3	2894

Search Date: 27-Mar-2025

In Web of Science, the initial search using Alzheimer's disease and CBT terms retrieved an overly broad result set. To improve specificity, we incorporated randomized controlled trial (RCT)-related terms—adopted from a validated search filter provided by the Hedges Project's Clinical Queries (Health Information Research Unit, McMaster University)—which focus on identifying RCTs. This refinement was made to enhance consistency across studies and to reduce unnecessary workload

Supplementary Figure S2. Funnel plots

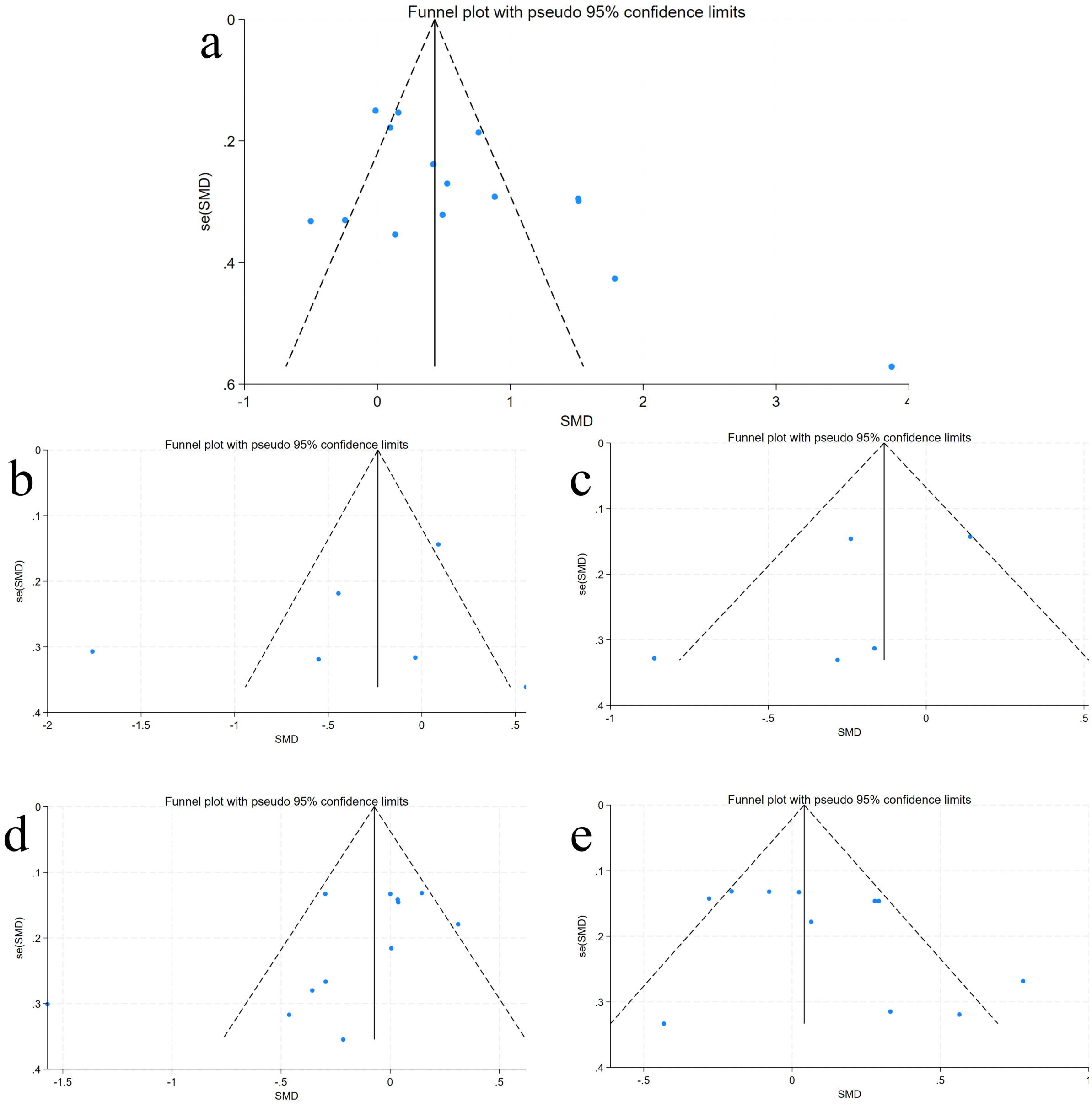

Notes: Funnel plots for the included outcomes. (a) MMSE; (b) CSDD; (c) GDS; (d) NPI; (e) QoL. Funnel plot analysis revealed that, with the exception of the GDS (Figure S2c), which exhibited noticeable asymmetry, the other four outcomes appeared largely symmetrical. This suggests a potential risk of publication bias for studies reporting GDS, whereas no obvious bias was detected for other outcomes.

Supplementary Figure S3. Sensitivity analyses

Supplementary Figure S3a. Sensitivity analyses for MMSE

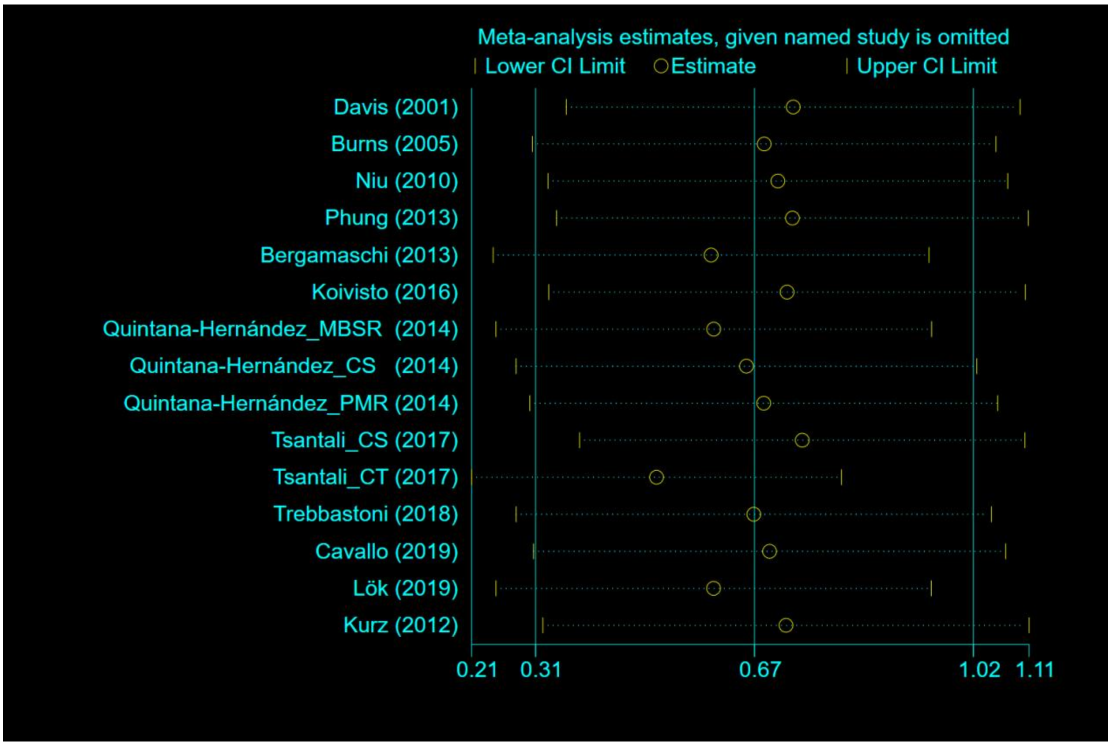

Notes: The leave-one-out sensitivity analysis for MMSE showed that the overall effect size remained stable when each study was excluded in turn. No individual study markedly altered the direction or significance of the pooled estimate, suggesting that the results are robust.

Supplementary Figure S3b. Sensitivity analyses for CSDD

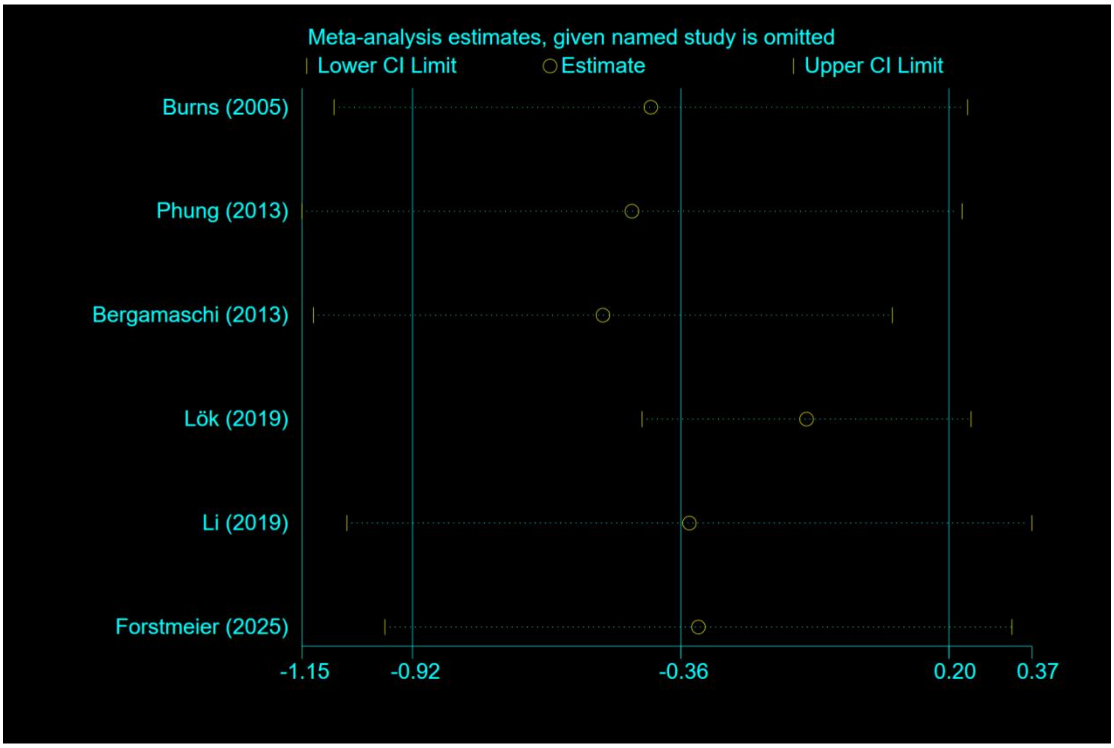

Notes: The pooled estimate remained consistent when each study was sequentially omitted, indicating no individual study had a disproportionate impact on the CSDD results.

Supplementary Figure S3c. Sensitivity analyses for GDS

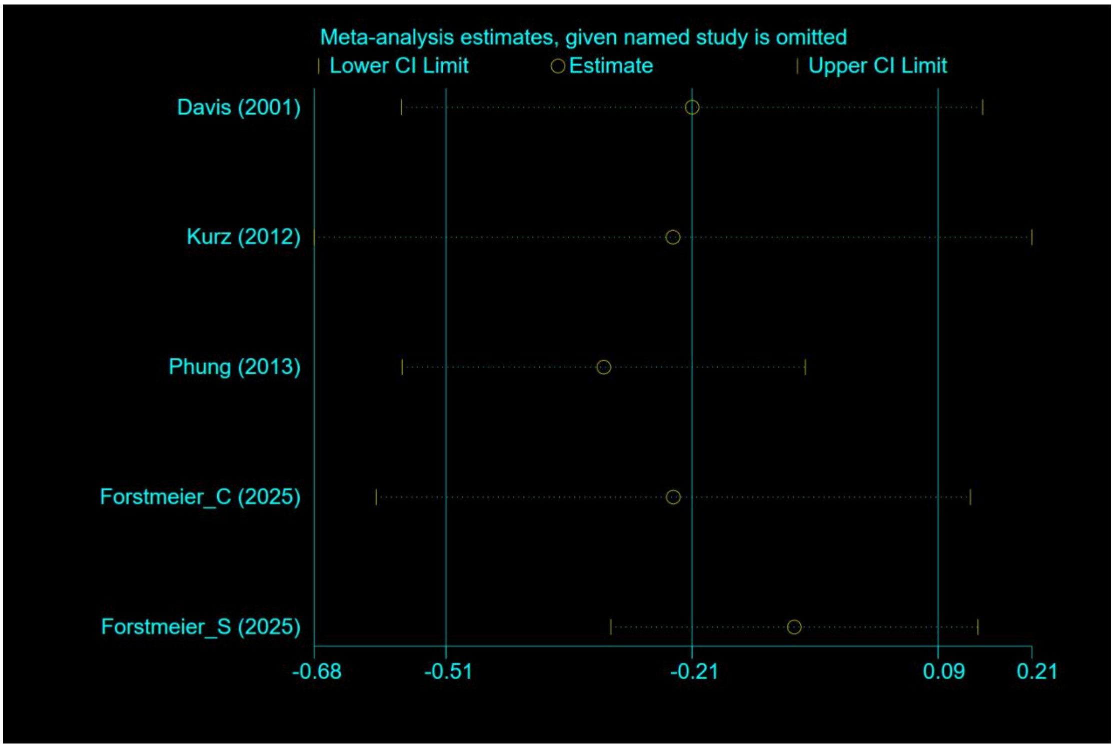

Notes: Excluding any single study did not substantially change the pooled effect size, suggesting that the results for GDS were robust.

Supplementary Figure S3d. Sensitivity analyses for NPI

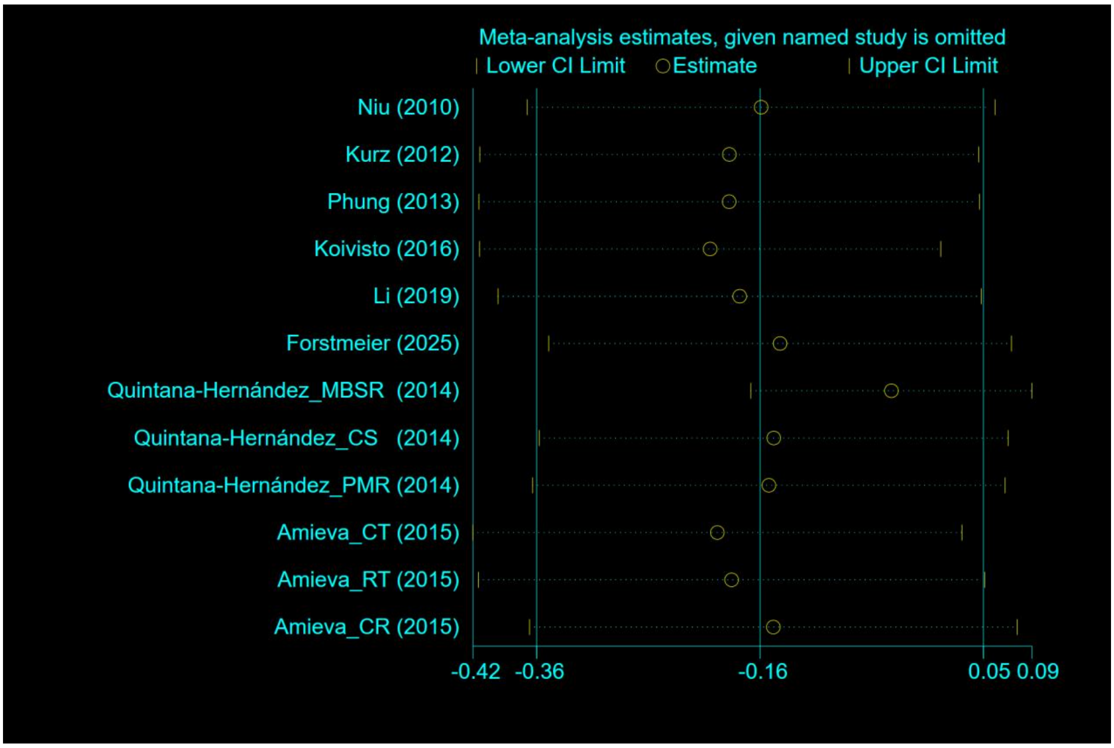

Notes: No marked changes in the pooled effect were observed when studies were removed one at a time, supporting the stability of the NPI findings.

Supplementary Figure S3e. Sensitivity analyses for QoL

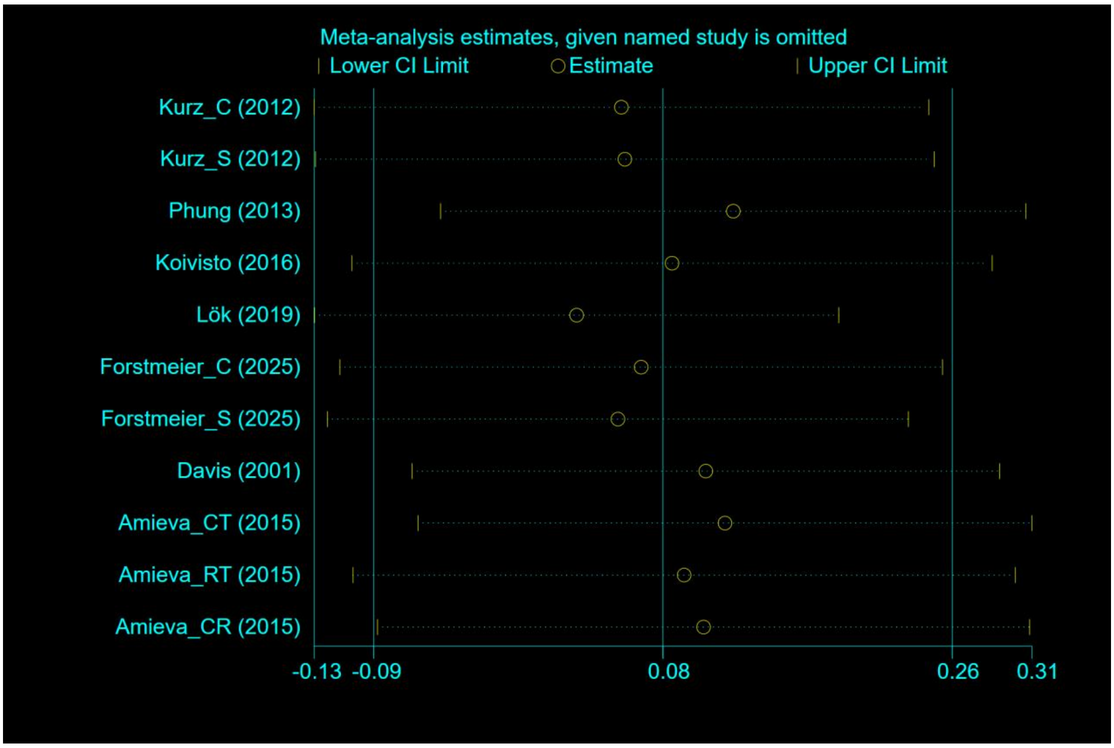

Notes: Sequential removal of studies did not significantly influence the overall pooled estimate, suggesting robustness of the QoL results.

Supplementary Figure S4. Subgroup analyses

Supplementary Figure S4a. Subgroup analysis for GDS

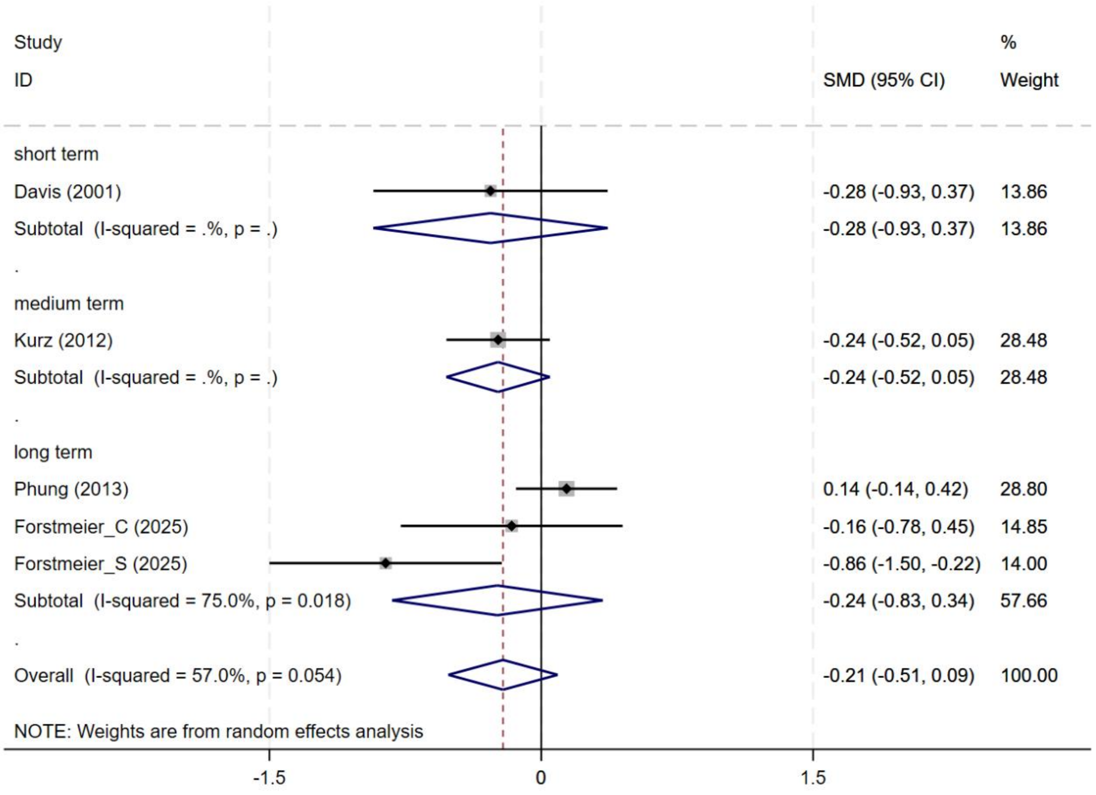

Subgroup analysis for GDS showed no significant difference.

Supplementary Figure S4b. Subgroup analysis for NPI

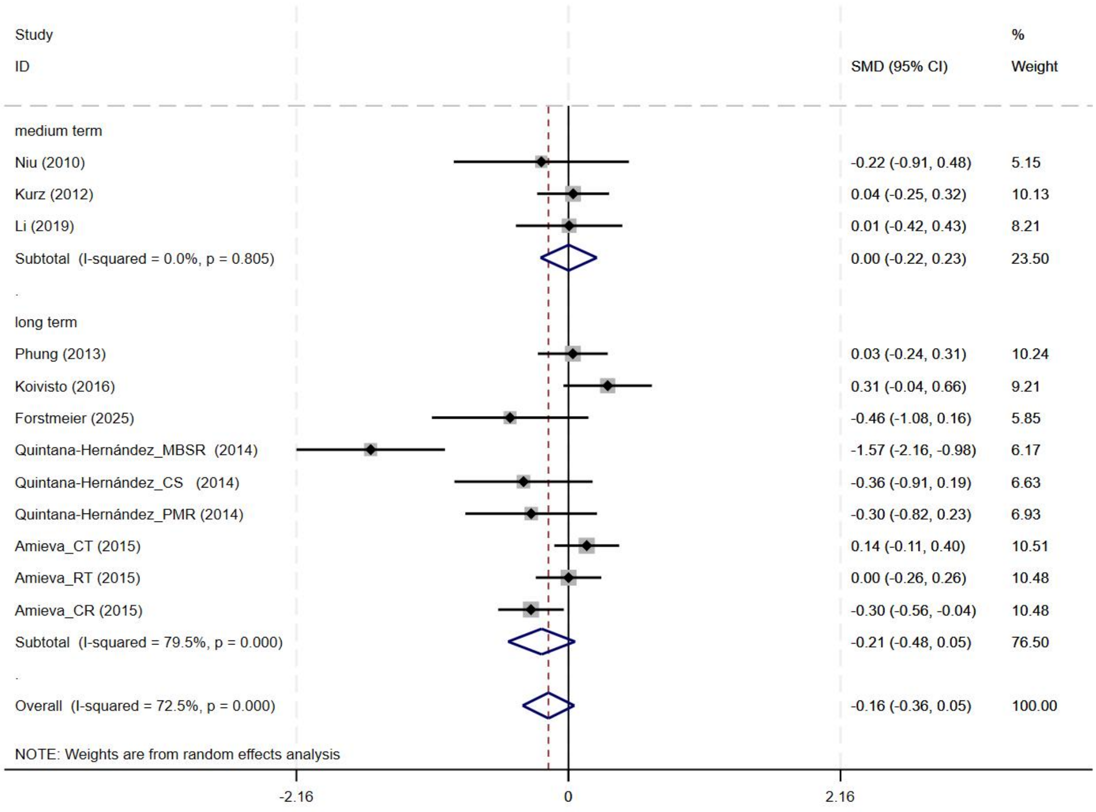

Subgroup analysis for NPI showed no significant difference

Supplementary Table S5. GRADE evidence summary

Outcome	No of studies	Sample size	Risk of bias	Inconsistency	Indirectness	Imprecision	Publication bias	Overall quality
MMSE	12	1132	Moderate	High heterogeneity subgroup: long-term CBT SMD=0.88, 95% CI 0.34 to 1.42	No concerns	No concerns	Some concerns	Low/Very low
CSDD	6	453	Moderate	High heterogeneity subgroup: medium-term CBT SMD=-.45,95% CI -0.87 to -0.02	No concerns	Some concerns	No concerns	Low
GDS	4	464	Moderate	Moderate heterogeneity	No concerns	Some concerns	Some concerns	Low/Moderate
NPI	8	1538	Moderate	High heterogeneity	No concerns	Some concerns	No concerns	Low
QoL	7	1128	Moderate	Moderate heterogeneity subgroup: medium-term CBT SMD=0.29,95%CI 0.08 to 0.49	No concerns	Some concerns	No concerns	Low/Moderate

Notes: The table summarizes 5 outcomes, MMSE (cognitive function), CSDD and GDS (depression), NPI (neuropsychiatric symptoms), and QoL (quality of life). Overall evidence quality ranged from low to moderate, mainly due to heterogeneity, Risk of bias, imprecision, and some concerns about publication bias.

Supplementary Table S6. Calculation of pooled SD for MMSE

The overall pooled SD was calculated according to conventional formulas:

Step 1: Calculation of pooled SD for each individual study

$$SD_{pooled,i} = \sqrt{\frac{(n_{exp} - 1) \times SD_{exp}^2 + (n_{ctrl} - 1) \times SD_{ctrl}^2}{n_{exp} + n_{ctrl} - 2}}$$

where:

n_{exp} = sample size of CBT group

n_{ctrl}= sample size of the control group

SD_{exp}=standard deviation of CBT group

SD_{ctrl}= standard deviation of the control group

Step 2: Calculation of the overall pooled SD across multiple studies

After obtaining the pooled SD for each individual study, the overall pooled SD across all included studies was calculated, weighting each study's SD according to its sample size.

$$SD_{overall} = \sqrt{\frac{\sum_i (n_i - 1) \quad SD_{pooled,i}^2}{\sum_i (n_i - 1)}}$$

Step 3:

The overall pooled SD calculated in this study was 4.2807. Multiplying this value by the standardized mean difference (SMD = 0.67) yielded an estimated absolute change of 2.87 points on the MMSE, suggesting potential clinical relevance:

$$\Delta MMSE \approx SMD \times SD_{overall} = 0.67 \times 4.28 \approx 2.87$$

Supplementary Table S7. Potential sources of heterogeneity

Source	Examples from included studies	Potential impact on results
Population	(a) Mean age ranged from 68 (Davis, 2001) to 83 years (Li, 2019);<br>(b) gender distribution varied (male/female 7/25 in Niu, 2010 vs. 151/179 in Phung, 2013);<br>(c) The severity of AD ranged from mild to moderate, consistent with its continuous disease course. Six studies exclusively recruited patients with mild AD (Burns, 2005; Kurz, 2012; Phung, 2013; Koivisto, 2015; Tsantali, 2017; Forstmeier, 2025).	Differences in baseline cognition, gender, and dementia severity may influence intervention effects and reduce comparability.
Intervention	(a) Session frequency varied from once weekly (Davis, 2001) to 5-6 times per week (Bergamaschi, 2013);<br>(b) Delivery format varied across studies, including individual(Cavallo 2018) and group interventions(Amieva,2016);	Inconsistent intervention intensity and delivery format may contribute to substantial heterogeneity in effect sizes.
Control condition	Controls varied: usual care (Kurz, 2012), non-specific cognitive activity (Bergamaschi, 2013), or no intervention (Trebastoni, 2018; Lök, 2019).	Differences in control conditions affect the relative magnitude of CBT effects.
Methodologic	Allocation concealment was inadequately addressed in most studies; participant blinding was implemented in only two trials.	This may increase risk of performance and detection bias, weakening the robustness of pooled results.
Sample size	Sample sizes ranged from 20 (Davis, 2001; Tsantali, 2017) to 330+ (Phung, 2013);	Small studies contribute to imprecision and may inflate heterogeneity.
Publication bias	Egger's test p=0.025 in MMSE suggested small-study effects;	Small positive trials more likely to be published, potentially overestimating pooled effects.

Notes: Comparisons across studies were conducted, and potential sources of heterogeneity were explored. High heterogeneity may be attributed to differences in study populations, intervention protocols, control conditions, methodological quality, sample size, and publication bias.
